# Using body composition to predict treatment-related adverse events and disease-free survival in patients with gastrointestinal stromal tumors treated with imatinib: a retrospective cohort study

**DOI:** 10.3389/fimmu.2025.1576834

**Published:** 2025-04-07

**Authors:** Tianhao Gu, Tengyun Li, Jianan Zhang, Qianzheng Zhou, Dinghua Yang, Jun Xu, Qiong Li, Zekuan Xu, Fengyuan Li, Hao Xu

**Affiliations:** ^1^ Department of General Surgery, The First Affiliated Hospital of Nanjing Medical University, Nanjing, China; ^2^ Department of Imaging, The First Affiliated Hospital of Nanjing Medical University, Nanjing, China; ^3^ Collaborative Innovation Center for Cancer Personalized Medicine, Nanjing Medical University, Nanjing, Jiangsu, China

**Keywords:** myosteatosis, CXI index, sarcopenia, treatment-related adverse events, disease-free survival, gastrointestinal stromal tumors

## Abstract

**Background:**

Imatinib (IM) is the primary treatment for Gastrointestinal stromal tumor (GIST), but it faces significant challenges with resistance and a high incidence of adverse events. This study aims to assess the predictive value of baseline body composition parameters on treatment-related adverse events and disease-free survival (DFS) in GIST patients treated with imatinib.

**Materials and Methods:**

A single-center retrospective analysis was conducted on 107 moderate or high-risk stratification GIST patients diagnosed from 2014 to 2020 at the First Affiliated Hospital of Nanjing Medical University. Body composition parameters, including skeletal muscle index (SMI), myosteatosis, cachexia index (CXI), and Fat-Free Mass (FFM), etc. were obtained using abdominal CT images and clinical data. Logistic and COX regression models were used to analyze the relationship between these indicators and treatment-related adverse events and DFS.

**Results:**

Multivariate analysis revealed that myosteatosis (OR=7.640, P<0.001) and drug dose (OR=1.349, P=0.010) were independent risk factors for adverse events, while a higher CXI (OR=0.983, P=0.017) was protective. Additionally, LAMA/SMA% (OR=1.072, P=0.028) was identified as an independent risk factor for dose-limiting toxicity (DLT). Independent predictors of DFS included sarcopenia (HR=3.067, P=0.013), myosteatosis (HR=6.985, P=0.024), risk stratification (HR=9.562, high-risk vs. moderate-risk, P=0.003), and C-KIT mutation (HR=3.615, C-KIT exon 9 mutation vs. 11, P=0.013).

**Conclusions:**

Baseline body composition parameters, particularly myosteatosis, effectively predict the adverse events and DFS in patients taking imatinib. Personalized treatment, such as targeted nutritional and exercise interventions, and close monitoring of patients with myosteatosis or sarcopenia can enhance compliance and improve survival rates.

## 1 Introduction

Gastrointestinal stromal tumors (GISTs) are the most common mesenchymal tumors of the digestive system (1). They are thought to originate from the interstitial cells of Cajal (ICC) and consist of spindle or epithelioid cells with the potential for multidirectional differentiation (2). These tumors exhibit diverse morphologies and complex biological behaviors. GISTs can develop at any site within the gastrointestinal tract, most frequently in the stomach (60-65%) and small intestine (20-25%). Smaller proportions are found in the rectum (5-15%) and esophagus (5%), with occasional cases in the colon, appendix, and gallbladder (1). Globally, the incidence of GISTs is approximately 0.01-0.02‰, with a prevalence of around 0.13-0.16%, showing significant regional differences (3). In China, the large population results in about 40,000 new GIST cases annually, posing a serious public health threat and a substantial societal burden (4).

Imatinib (IM) is the first-line treatment for advanced or metastatic GIST and is considered the most effective targeted therapy available (5). Extensive multicenter clinical studies have demonstrated that IM primarily exerts a cytostatic effect, with only about 5% of GIST patients achieving complete clinical remission. Additionally, approximately 50% of GIST patients develop secondary resistance to IM after two years of treatment, and most advanced-stage patients eventually experience secondary resistance despite initial benefits from IM therapy (6, 7). Moreover, GIST cells are often still detectable in patients who have undergone tumor resection followed by IM treatment, indicating that secondary resistance to IM is very common in clinical practice.

In phase I, II, and III clinical trials of imatinib, nearly all patients experienced at least one adverse event of any grade (8–10). The most common non-hematologic adverse events included periorbital edema, diarrhea, nausea and vomiting, rash, and fatigue. The most frequent hematologic adverse events were anemia, neutropenia, thrombocytopenia, and elevated liver transaminases (11, 12). Although imatinib is generally considered a well-tolerated tyrosine kinase inhibitor (TKI), a significant proportion of patients experience substantial clinical toxicity, leading to frequent treatment interruptions.

Changes in body composition are common in patients with malignancies and are often linked to poor prognosis (13). Sarcopenia, an age-related condition, involves accelerated muscle mass loss, decreased strength, and reduced physical function. It has recently been identified as a predictor of poor disease-free survival (DFS) and overall survival (OS) in many cancers (14–16). Additionally, myosteatosis, the infiltration of fat into muscle, is associated with declines in muscle strength and quality (17). This condition has emerged as a potential predictor of treatment-related adverse events and survival rates in cancer patients (18, 19). The cachexia index (CXI) is a new measure that captures key features of cachexia, including reduced muscle mass, poor nutritional status, and systemic inflammation (20). CXI can also predict adverse treatment responses and overall prognosis (21).

Given the high incidence of resistance and adverse reactions associated with imatinib, it is essential to identify specific indicators that can predict toxic reactions and disease-free survival. Therefore, this study aims to investigate the role of baseline body composition parameters, including sarcopenia, myosteatosis, and the cachexia index, in predicting the incidence of toxic reactions and DFS in GIST patients treated with imatinib.

## 2 Materials and methods

The research protocol has been registered with the Chinese Clinical Trial Registry under registration number ChiCTR2400090130. It adhered to the Declaration of Helsinki, and ethical approval for this study (Ethical Committee 2024-SR-556) was provided by the Ethics Committee of the First Affiliated Hospital of Nanjing Medical University on July 1, 2024. In compliance with the committee’s requirements, all patients provided verbal informed consent to participate in the study.

The reporting of this single-center retrospective observational cohort study fulfills the STROCSS (Strengthening the Reporting of Cohort, cross-sectional and case–control Studies in Surgery) criteria (22). The pathology report of GIST specimens includes details on tumor site, tumor size, cell morphology, immunohistochemical analysis, mitotic rate, and risk stratification. For a comprehensive overview, please refer to the results section.

### 2.1 Study population and data collection

**Figure 1:**
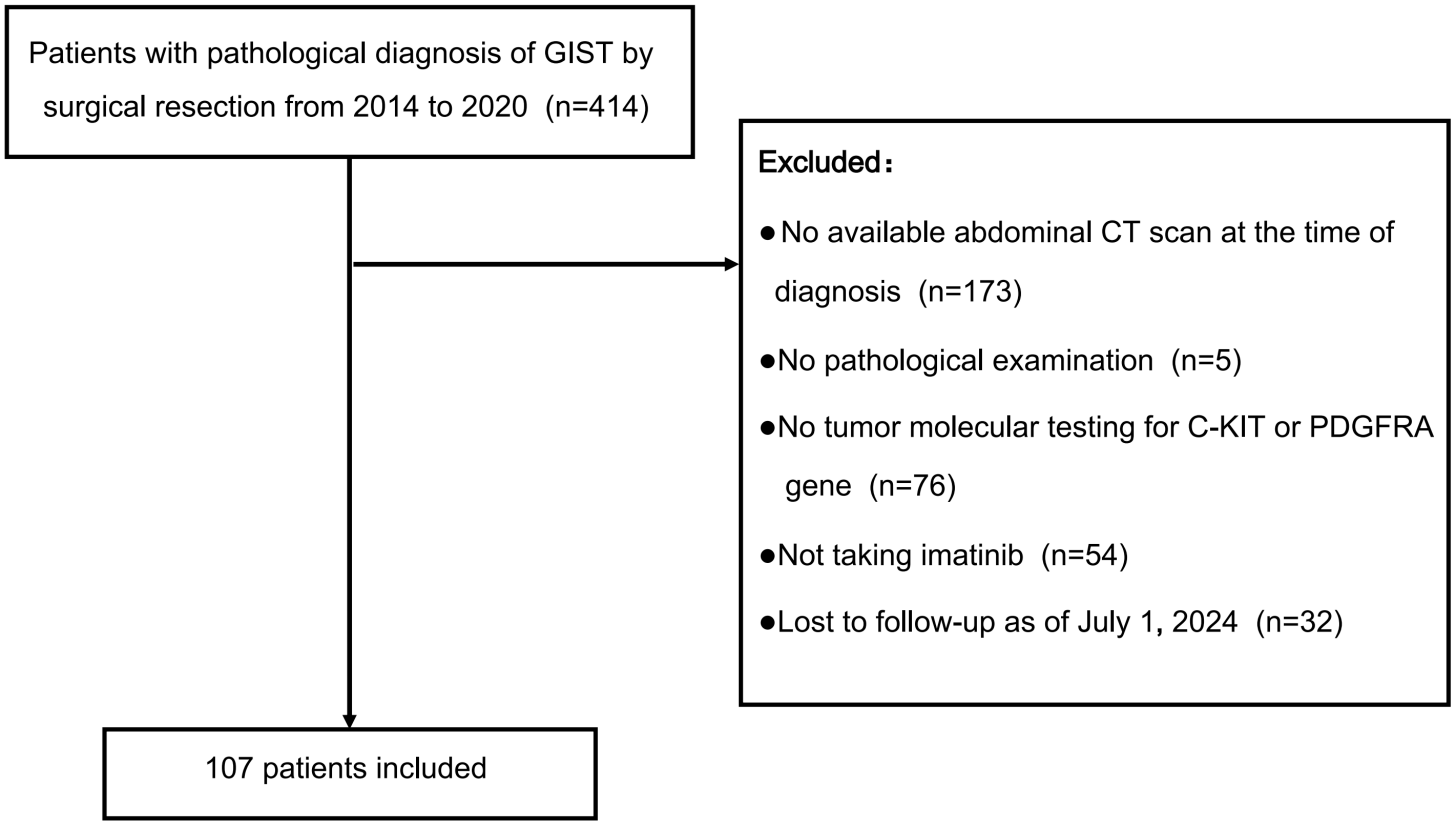
Selection of patients for the study. This flowchart details the selection process of 107 patients from an initial cohort of 414 diagnosed with GIST via surgical resection between 2014 and 2020, after excluding those without an abdominal CT scan, pathological examination, tumor molecular testing, imatinib treatment, or follow-up as of July 1, 2024.

This study is a single-center retrospective analysis conducted at the Department of General Surgery, First Affiliated Hospital of Nanjing Medical University. Between 2014 and 2020, we screened 414 patients with pathologically confirmed moderate or high-risk stratification GIST who had undergone surgical resection. The inclusion criteria were: (1) Age between 18 and 80 years. (2) Pathologically confirmed GIST, classified as moderate or high risk stratification according to the modified NIH classification (23). (3) Availability of C-KIT and PDGFR (Platelet-Derived Growth Factor Receptor) gene testing on surgically resected specimens. Exclusion criteria included: (1) Lack of an abdominal computed tomography (CT) scan within one month prior to diagnosis or medication. (2) Insufficient clinicopathological or genetic testing data. (3) Absence of imatinib treatment post-diagnosis. (4) Presence of other concurrent tumors. (5) Loss of contact or follow-up. After applying these criteria, 107 patients were included in the final analysis (**Figure 1**).

All patients were followed up postoperatively via telephone interviews, outpatient visits, and the Chinese social media application WeChat. The final follow-up date was July 1, 2024. At the last follow-up, patients were classified as either experiencing recurrence or remaining healthy. Disease-free survival was defined as the time from the initial surgery to either disease recurrence or death. For the DFS analysis, only “recurrence” was counted as an event.

### 2.2 Body composition analysis

**Figure 2:**
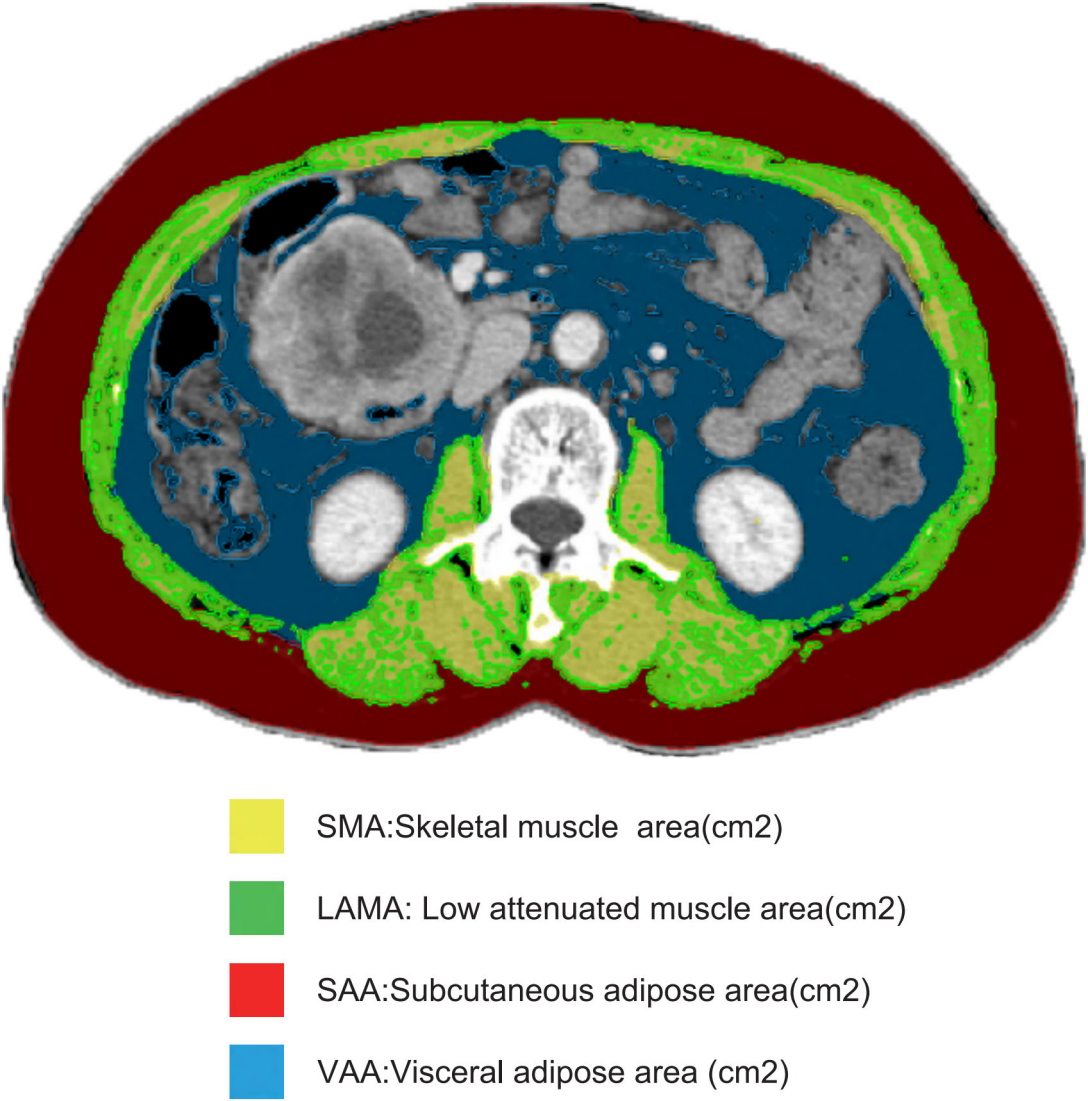
Body composition measurements. This figure presents the measurements of skeletal muscle area (SMA), low attenuated muscle area (LAMA), subcutaneous fat area (SAA), and visceral fat area (VAA) on a single CT image of the third lumbar vertebra (L3), all measured in square centimeters (cm²), used to assess body composition in the study.


Body composition consists of both fat and non-fat tissues. Fat tissue includes subcutaneous fat, visceral fat, and intermuscular fat, while non-fat tissue comprises muscle, bone, and internal organs. A single CT image at the third lumbar vertebra (L3) is commonly used to quantify muscle and fat characteristics due to its anatomical correlation with body volume (24). We employed the Siemens Healthineers MM Radiomics software to capture accurate images at L3, manually delineating regions of interest to differentiate between muscle and fat tissues. Using standard Hounsfield unit (HU) ranges, we quantified the cross-sectional area of skeletal muscle tissue (SMT, -29 to 150 HU), visceral adipose tissue (VAT, -150 to -50 HU), and subcutaneous adipose tissue (SAT, -190 to -30 HU) (**Figure 2**). Each body composition measurement (cm²) was then divided by height squared (m²) to convert it into an index: skeletal muscle index (SMI), visceral adipose index (VAI), and subcutaneous adipose index (SAI) (25).

$$Skeletal\;Muscle\;Index[SMI]=Skeletal\;muscle\;area[SMA](cm^{2})/Height^{2}(m^{2})$$

$$Visceral\;Adipose\;Index[VAI]=Visceral\;adipose\;area[VAA](cm^{2})/Height^{2}(m^{2})$$

$$Subcutaneous\;Adipose\;Index[SAI]=Subcutaneous\;adipose\;area[SAA](cm^{2})/Height^{2}(m^{2})$$

Low attenuation muscle area (LAMA) is measured using a predefined threshold range of -29 to +29 HU for new skeletal muscle area (**Figure 2**). The LAMA/SMA index is expressed as a percentage: 100% × (LAMA (cm²)/SMA (cm²)). Patients with a LAMA/SMA index ≥ 20% are considered to have myosteatosis (19).

We established sex-specific L3 SMI cut-off values using the Youden index method, based on the most significant survival differences. For females, the SMI cut-off value is 38.31 cm²/m², while for males, it is 44.53 cm²/m². An SMI below these thresholds indicates sarcopenia (26). Similarly, we defined sex-specific cut-off values for VAI and SAI. The VAI cut-off values are 37.56 cm²/m² for females and 22.66 cm²/m² for males. The SAI cut-off values are 91.44 cm²/m² for females and 20.42 cm²/m² for males.

Body Mass Index (BMI) estimates body fat for individuals of any age and is calculated as BMI = weight (kg)/height² (m²). According to the WHO (27), a BMI of 18.5-24.9 kg/m² is considered normal weight, while a BMI of 25 kg/m² or higher is classified as overweight or obese.

Fat-Free Mass (FFM) includes all non-fat tissues, such as skeletal muscle mass, metabolic organs like the liver and kidneys, intracellular and extracellular water, and bone tissue, accounting for most resting energy expenditure (28). The whole-body fat-free mass is estimated using the regression equation described by *Mourtzakis* (24): Whole-body fat-free mass (kg) = 0.3 × skeletal muscle at L3 (cm²) + 0.06. Drug dose is expressed per FFM (e.g., mg of imatinib/kg FFM), and the association of this variable with toxicity is evaluated.

### 2.3 Cachexia index

The cachexia index (CXI) is calculated using the formula: CXI = SMI × Alb/NLR. In this formula, SMI stands for the skeletal muscle index, Alb represents the serum albumin measured in g/dL, and NLR is the neutrophil-to-lymphocyte ratio, which is calculated by dividing the absolute neutrophil count by the absolute lymphocyte count (21). Using the Youden index method, we established a CXI cut-off value of 48.57, which showed the most significant survival differences.

### 2.4 Adverse events

Treatment-related adverse events (TRAEs) were evaluated using the Common Terminology Criteria for Adverse Events (CTCAE) version 5.0 from the National Cancer Institute (29). TRAEs encompassed all events reported from the first dose to the last follow-up. For further analysis, toxicity was classified into grades I-II and III-IV. Dose-limiting toxicity (DLT) was defined as any toxicity that necessitated dose reduction or led to temporary or permanent discontinuation of treatment. This included grade III non-hematologic toxicity, grade IV hematologic toxicity, and certain grade II toxicities such as nephrotoxicity and cardiotoxicity (30).

### 2.5 Statistical analysis

Normally distributed variables are presented as means and standard deviations, while categorical variables are described using absolute numbers and percentages. Pearson’s chi-squared test or Fisher’s exact test was used to compare categorical variables, and Student’s t-test or the Wilcoxon rank-sum test was used for continuous variables. Logistic regression models were utilized for both univariate and multivariate analyses to examine the relationships between various indices and treatment-related adverse events. The Hosmer-Lemeshow test assessed the goodness of fit for logistic regression models, and the Variance Inflation Factor (VIF) detected multicollinearity in regression analyses. For high-dimensional data processing and variable selection, the least absolute shrinkage and selection operator (LASSO) regression technique was applied. Internal validation was conducted using the bootstrap method with 1,000 resamples. A nomogram was developed using multivariate logistic regression analysis, and its predictive performance was evaluated with ROC curves and calibration curves. Decision curve analysis (DCA) was used to assess the nomogram’s clinical utility. To analyze the relationships between various indices and disease-free survival, use Cox proportional hazards regression models for univariate and multivariate analyses. Kaplan-Meier survival analysis of DFS was conducted with the log-rank test. A nomogram for predicting DFS was created using multivariate Cox regression analysis. Statistical significance was set at p < 0.05. All statistical analyses were performed using R version 4.4.0 (R Foundation for Statistical Computing, Vienna, Austria) and SPSS version 26.0, RRID: SCR_002865 (IBM Corp., Armonk, NY, USA).

## 3 Results

### 3.1 Patient characteristics

The baseline characteristics of 107 patients are detailed in **Table 1**. The average age of the cohort was 58.96 years, with an average BMI of 23.74 kg/m² and an average LAMA/SMA% of 26.99%. Myosteatosis was identified in 74 patients (69.16%). Adverse events of any grade during imatinib treatment were experienced by 78 patients (72.90%), with dose-limiting toxicity observed in 27 patients (25.25%). There were no significant differences between males and females in terms of tumor location, risk classification, or C-KIT and PDGFR gene mutation types. However, significant gender differences were found in the incidence of adverse events and specific adverse events such as edema, skin rash, granulocytopenia, and anemia. Regarding body composition indicators, males and females showed significant differences in SMA, SAA, SMI, and SAI (p < 0.001). Due to the lack of internationally recognized cut-off values for SMI, we established sex-specific cut-off values for SMI, SAI, and VAI, based on the most significant survival differences.

**Table 1:** Baseline characteristics of the patients.

Characteristic	Overall, N = 107	Female, N = 47	Male, N = 60	p-value^a^
Age, Mean (SD)	58.96 (10.81)	59.70 (11.59)	58.38 (10.22)	0.528
Serum albumin, Mean (SD)	38.73 (5.20)	39.54 (4.69)	38.10 (5.52)	0.083
Neutrophils, Mean (SD)	4.01 (1.74)	3.73 (1.63)	4.22 (1.80)	0.221
Lymphocyte, Mean (SD)	1.50 (0.58)	1.51 (0.55)	1.50 (0.61)	0.737
Height, Mean (SD)	1.65 (0.07)	1.59 (0.05)	1.70 (0.05)	<0.001
Weight, Mean (SD)	64.79 (10.07)	58.54 (9.30)	69.69 (7.70)	<0.001
BMI, Mean (SD)	23.74 (3.07)	23.20 (3.59)	24.17 (2.54)	0.054
Tumor site, n (%)				0.519
Gastric	53.00 (49.53%)	27.00 (57.45%)	26.00 (43.33%)	
Duodenum	14.00 (13.08%)	3.00 (6.38%)	11.00 (18.33%)	
Jejunum/ileum	27.00 (25.23%)	12.00 (25.53%)	15.00 (25.00%)	
Colon	1.00 (0.93%)	0.00 (0.00%)	1.00 (1.67%)	
Rectum	2.00 (1.87%)	1.00 (2.13%)	1.00 (1.67%)	
Abdominal	5.00 (4.67%)	2.00 (4.26%)	3.00 (5.00%)	
Pelvic	1.00 (0.93%)	1.00 (2.13%)	0.00 (0.00%)	
Liver	1.00 (0.93%)	0.00 (0.00%)	1.00 (1.67%)	
Unknown	3.00 (2.80%)	1.00 (2.13%)	2.00 (3.33%)	
Risk stratification, n (%)				0.107
Moderate	33.00 (30.84%)	19.00 (40.43%)	14.00 (23.33%)	
High	62.00 (57.94%)	22.00 (46.81%)	40.00 (66.67%)	
Unknown	12.00 (11.21%)	6.00 (12.77%)	6.00 (10.00%)	
C-KIT mutations, n (%)				>0.999
Exon 9	12.00 (11.21%)	5.00 (10.64%)	7.00 (11.67%)	
Exon 11	86.00 (80.37%)	39.00 (82.98%)	47.00 (78.33%)	
Exon 13	3.00 (2.80%)	1.00 (2.13%)	2.00 (3.33%)	
Exon 11 + 13	1.00 (0.93%)	0.00 (0.00%)	1.00 (1.67%)	
Exon 9 + 11	1.00 (0.93%)	0.00 (0.00%)	1.00 (1.67%)	
Wild	4.00 (3.74%)	2.00 (4.26%)	2.00 (3.33%)	
PDGFR mutations, n (%)				>0.999
Exon 18	1.00 (0.93%)	0.00 (0.00%)	1.00 (1.67%)	
Wild	106.00 (99.07%)	47.00 (100.00%)	59.00 (98.33%)	
Adverse events, n (%)	78.00 (72.90%)	42.00 (89.36%)	36.00 (60.00%)	<0.001
Dose-limiting toxicity, n (%)	27.00 (25.23%)	16.00 (34.04%)	11.00 (18.33%)	0.063
Edema, n (%)	55.00 (51.40%)	30.00 (63.83%)	25.00 (41.67%)	0.023
Skin rash, n (%)	35.00 (32.71%)	17.00 (36.17%)	18.00 (30.00%)	0.500
Granulocytopenia, n (%)	26.00 (24.30%)	17.00 (36.17%)	9.00 (15.00%)	0.011
Anemia, n (%)	14.00 (13.08%)	13.00 (27.66%)	1.00 (1.67%)	<0.001
Thrombocytopenia, n (%)	1.00 (0.93%)	1.00 (2.13%)	0.00 (0.00%)	0.439
Nausea and vomiting, n (%)	6.00 (5.61%)	4.00 (8.51%)	2.00 (3.33%)	0.401
Diarrhea, n (%)	1.00 (0.93%)	0.00 (0.00%)	1.00 (1.67%)	>0.999
Dyspepsia, n (%)	5.00 (4.67%)	3.00 (6.38%)	2.00 (3.33%)	0.652
Liver dysfunction, n (%)	2.00 (1.87%)	0.00 (0.00%)	2.00 (3.33%)	0.503
SMA, Mean (SD)	125.09 (37.63)	97.30 (14.07)	146.86 (35.95)	<0.001
SAA, Mean (SD)	119.28 (54.09)	142.68 (63.53)	100.95 (36.51)	<0.001
VAA, Mean (SD)	102.85 (60.26)	94.30 (51.68)	109.55 (65.86)	0.290
LAMA, Mean (SD)	32.65 (13.71)	30.00 (9.77)	34.72 (15.92)	0.265
SMI, Mean (SD)	45.54 (11.84)	38.57 (5.61)	51.00 (12.58)	<0.001
SAI, Mean (SD)	44.42 (21.15)	56.22 (23.60)	35.18 (13.11)	<0.001
VAI, Mean (SD)	37.77 (21.53)	37.45 (20.31)	38.01 (22.61)	0.918
LAMA/SMA %, Mean (SD)	26.99 (10.25)	31.01 (9.71)	23.84 (9.60)	<0.001
Myosteatosis, n (%)	74.00 (69.16%)	42.00 (89.36%)	32.00 (53.33%)	<0.001
CXI, Mean (SD)	75.36 (39.13)	74.06 (41.84)	76.38 (37.21)	0.587
FFM, Mean (SD)	37.59 (11.29)	29.25 (4.22)	44.12 (10.79)	<0.001
Drug dose, Mean (SD)	11.19 (2.83)	13.40 (2.52)	9.46 (1.58)	<0.001

BMI, Body Mass Index; SMA, Skeletal Muscle Area; SAA, Subcutaneous Adipose Area; VAA, Visceral Adipose Area; LAMA, Low Attenuation Muscle Area; SMI, Skeletal Muscle Index; SAI, Subcutaneous Adipose Index; VAI, Visceral Adipose Index; CXI, Cachexia Index; FFM, Fat-Free Mass.

a Wilcoxon rank sum test; Fisher’s exact test; Pearson’s Chi-squared test.

### 3.2 Association between various indices and treatment-related adverse events

#### 3.2.1 Adverse events

**Supplementary Table S1** presents the results of the univariate logistic regression analysis for various indices and their association with the occurrence of any adverse events. The potential indicators included in the multivariate logistic analysis were sex (P=0.001), SMA (P=0.016), VAA (P=0.040), SMI (P=0.019), LAMA/SMA% (P=0.001), myosteatosis (P<0.001), CXI (P=0.011), FFM (P=0.016), and drug dose (P=0.001). The Hosmer-Lemeshow test P-value for VAA in the univariate logistic regression was 0.0075, indicating a poor fit for the logistic regression model, thus VAA was excluded from the multivariate regression analysis. Due to multicollinearity among SMA, FFM, and SMI (VIF>10), SMI was chosen for the multivariate logistic regression analysis as it is more clinically relevant. Similarly, due to multicollinearity between LAMA/SMA% and myosteatosis (VIF>10), two separate models were constructed for these variables. **Supplementary Table S2**, which included sex, SMI, myosteatosis, CXI, and drug dose in the multivariate logistic analysis, showed a Hosmer-Lemeshow test P-value of 0.880. **Supplementary Table S3**, which included sex, SMI, LAMA/SMA%, CXI, and drug dose, had a Hosmer-Lemeshow test P-value of 0.040. The model in **Supplementary Table S2** demonstrated a better fit. The final variables selected for the multivariate logistic analysis of any adverse events were myosteatosis (P=0.001), CXI (P=0.014), and drug dose (P=0.048).

**Figure 3:**
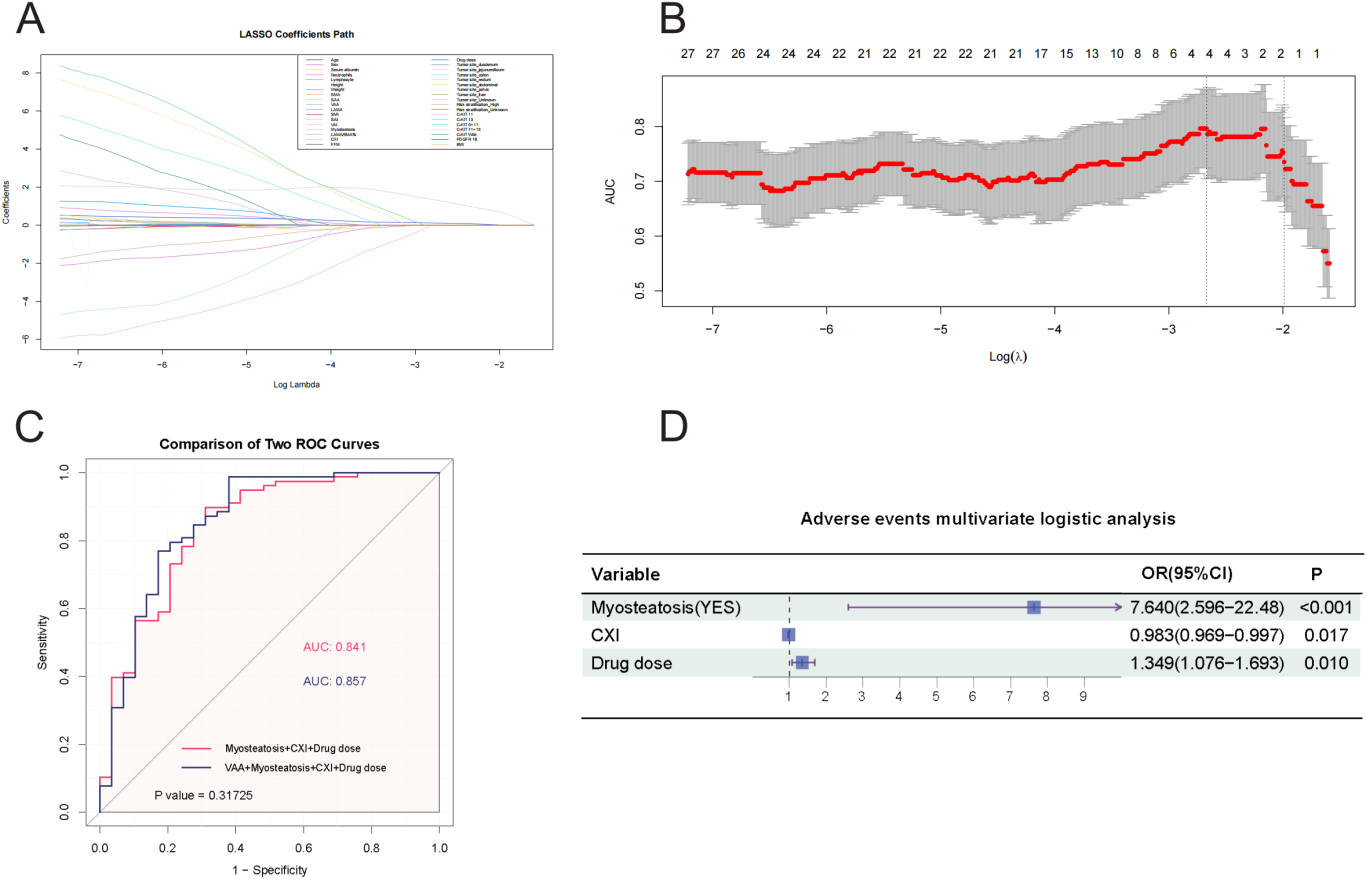
**(A)** LASSO Regression Coefficient Path. This figure illustrates the paths of regression coefficients for various predictors of adverse events as a function of the logarithm of the regularization parameter (Log Lambda) in a LASSO regression, demonstrating how the LASSO method shrinks some coefficients to zero for variable selection while estimating others, with each line representing a predictor’s coefficient trajectory and key values of Log Lambda marked to indicate when predictors enter or exit the model. **(B)** AUC vs. Log Lambda in LASSO Regression. This figure displays the relationship between the Area Under the Curve (AUC) and the logarithm of the regularization parameter (Log Lambda) in a LASSO regression model, showing how AUC values change with varying regularization strength and indicating the number of predictors of adverse events that remain at each level, thereby highlighting the optimal Log Lambda range for the best balance between model complexity and predictive performance. **(C)** Comparison of ROC Curves for Predicting Adverse Reactions. This figure compares two ROC curves for predicting adverse events: Model 1, which uses Myosteatosis, CXI, and Drug dose as predictors (AUC = 0.841), and Model 2, which adds VAA to the predictors (AUC = 0.857). The statistical comparison yields a P value of 0.31725. The X-axis represents 1 − Specificity (False Positive Rate), and the Y-axis represents Sensitivity (True Positive Rate). **(D)** A forest plot. This forest plot summarizes the multivariate logistic regression analysis for predicting adverse events, with the horizontal axis representing odds ratios (OR) on a logarithmic scale and each variable plotted as a point estimate with 95% confidence intervals.

To ensure the accuracy of the selected variables, we included all variables in the LASSO regression analysis (**Figures 3A, B**), which identified four variables with the highest AUC value: VAA, myosteatosis, CXI, and drug dose. **Supplementary Table S4**, which included myosteatosis, CXI, and drug dose in the multivariate logistic analysis, showed a Hosmer-Lemeshow test P-value of 0.356. **Supplementary Table S5**, which included VAA, myosteatosis, CXI, and drug dose, had a Hosmer-Lemeshow test P-value of 0.036. Although the AUC values of the ROC curves for the two models were similar (**Figure 3C**), the model including VAA had a Hosmer-Lemeshow test P-value < 0.05, indicating a poor fit. Therefore, the final multivariate logistic regression analysis for adverse events included myosteatosis, CXI, and drug dose (**Figure 3D**). The odds ratio (OR) for myosteatosis was 7.640 (95% CI: 2.596-22.48), for CXI was 0.983 (95% CI: 0.969-0.997), and for drug dose was 1.349 (95% CI: 1.076-1.693). These results indicate that myosteatosis and drug dose are independent risk factors for adverse events, while a higher CXI is a protective factor.

**Figure 4:**
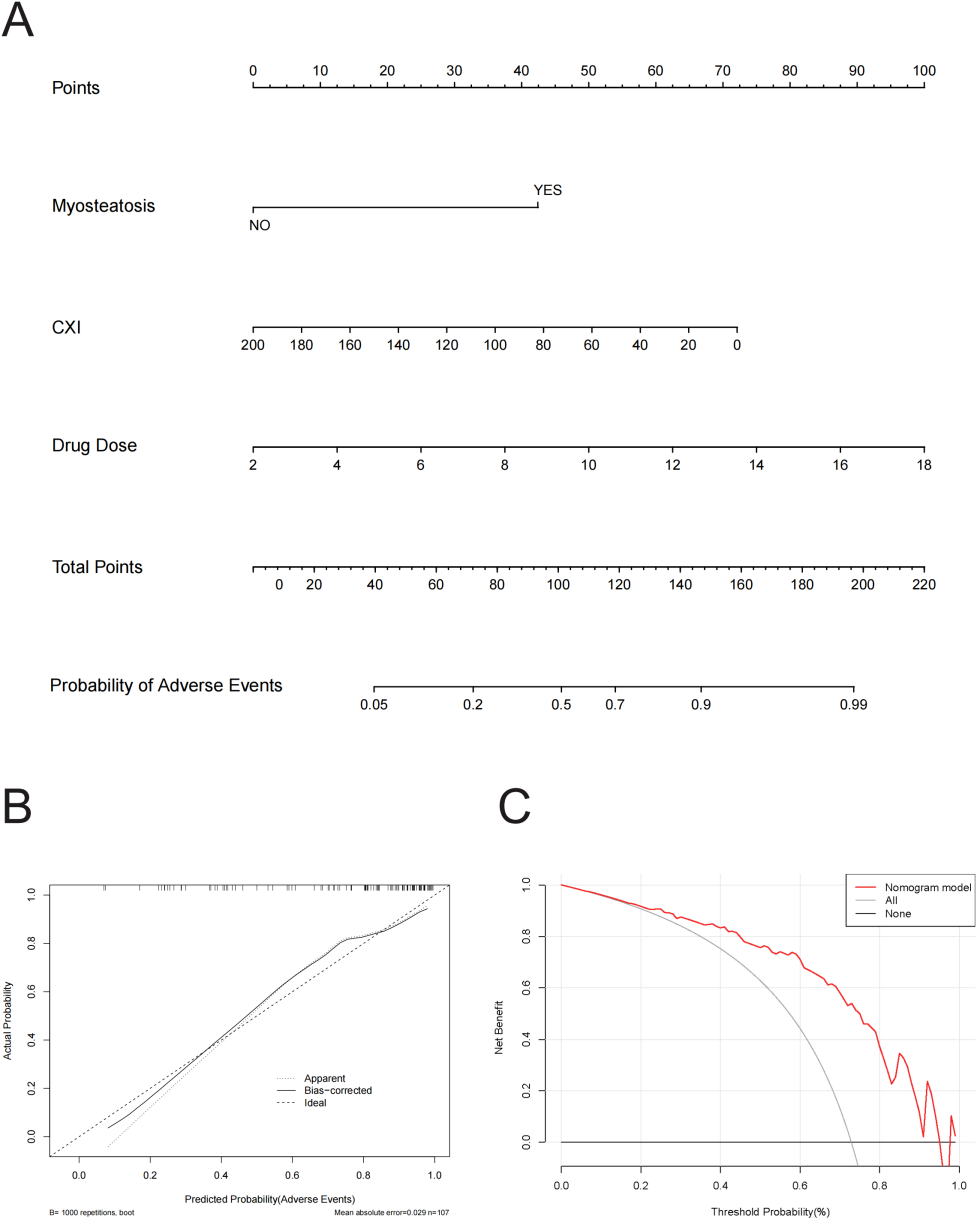
**(A)** Nomogram for Predicting Probability of adverse events. This nomogram integrates three variables—Myosteatosis, CXI, and Drug Dose—to predict the probability of adverse events, where each variable is assigned points, summed to a total score, and then mapped to a corresponding probability, providing a visual tool for clinicians to estimate individual patient risk. **(B)** Calibration Curve for Predicted vs. Actual Probability of Adverse Events. This calibration curve shows the relationship between predicted and actual probabilities of adverse events, with lines representing the apparent fit, bias-corrected fit (using 1000 bootstrap repetitions), and the ideal perfect calibration, and indicates a mean absolute error of 0.029 based on 107 observations. **(C)** Decision Curve Analysis (DCA) for Predicting Adverse Events. This figure presents the Decision Curve Analysis demonstrating the net benefit of the nomogram model for predicting adverse events across a range of threshold probabilities, compared to strategies of treating all patients or none, illustrating the model’s potential clinical utility.

To illustrate the predictive model for adverse events, we constructed a nomogram, which provides a convenient personalized tool to predict the probability of adverse events (**Figure 4A**). The proposed model demonstrated good calibration, showing a high degree of consistency between predicted probabilities and observed outcomes (**Figure 4B**). To evaluate its clinical utility, we conducted a decision curve analysis. The decision curve indicated that, using this nomogram, the threshold probability range for predicting adverse events was 0.17 to 0.97 (**Figure 4C**). Within this range, the nomogram offered more benefit compared to the “treat all” or “treat none” approaches.

#### 3.2.2 Dose-limiting toxicity

The univariate analysis results for dose-limiting toxicity are shown in **Supplementary Table S6**. Due to multicollinearity among SMA, FFM, and SMI (VIF > 10), SMI was selected for the multivariate logistic regression analysis as it is more clinically meaningful. Similarly, due to multicollinearity between LAMA/SMA% and myosteatosis (VIF > 10), we constructed two separate multivariate logistic regression models. **Supplementary Table S7** included age, height, weight, SMI, myosteatosis, CXI, and drug dose, showing a Hosmer-Lemeshow test P-value of 0.965, indicating a good fit. **Supplementary Table S8** included age, height, weight, SMI, LAMA/SMA%, CXI, and drug dose, with a Hosmer-Lemeshow test P-value of 0.687, also demonstrating a good fit. However, in the multivariate regression analysis of **Supplementary Table S7**, all variables had P-values > 0.05, indicating no significant predictors. In contrast, in **Supplementary Table S8**, LAMA/SMA% had a P-value of 0.028, with an OR of 1.072 (95% CI: 1.007-1.141), identifying it as a significant predictor of DLT.

To ensure the accuracy of the selected variables, we included all variables in the LASSO regression analysis (**Supplementary Figure S1**). This analysis identified LAMA/SMA% as the variable with the highest AUC value. Consequently, LAMA/SMA% was determined to be an independent risk factor for the occurrence of dose-limiting toxicity.

#### 3.2.3 Edema

The univariate analysis results for edema are shown in **Supplementary Table S9**. Due to multicollinearity among LAMA, LAMA/SMA%, and myosteatosis (VIF > 10), two separate models were constructed for comparison, focusing on LAMA/SMA% and myosteatosis (**Supplementary Tables S10, S11**). Variables with P < 0.05 in these models were selected for further multivariate logistic regression analysis (**Supplementary Tables S12, S13**). The model including myosteatosis had a Hosmer-Lemeshow test P-value of 0.134, while the model including LAMA/SMA% had a Hosmer-Lemeshow test P-value of 0.05. Therefore, we selected absolute neutrophil count (P=0.001) and myosteatosis (P<0.001) from the model in **Supplementary Table S12** as variables for the multivariate regression analysis.

To ensure the accuracy of the selected variables, all variables were included in the LASSO regression analysis (**Supplementary Figure S2A, B**), which identified CXI (P<0.001) and myosteatosis (P=0.001) as the two variables with the highest AUC values (**Supplementary Table S14**). Since the two methods selected different variables, we compared the models including absolute neutrophil count and myosteatosis with the model including CXI and myosteatosis (**Supplementary Figure S2C**). The model including CXI and myosteatosis had a higher AUC value (0.777 vs. 0.770). Although there was no significant statistical difference in the AUC values between the two models, the CXI formula includes the absolute neutrophil count, and CXI has greater clinical significance than the absolute neutrophil count. Therefore, in the final multivariate logistic regression analysis for edema, we selected CXI and myosteatosis as the variables.

Similarly, to illustrate the predictive model for the specific adverse event of edema, we constructed a nomogram (**Supplementary Figure S2D**) to provide a convenient personalized tool for predicting the probability of edema occurrence. The proposed model demonstrated good calibration (**Supplementary Figure S2E**). To evaluate its clinical utility, decision curve analysis was performed. The decision curve indicated that within a threshold probability range of 0.21 to 0.87, using this nomogram to predict the incidence of edema would yield the greatest net benefit (**Supplementary Figure S2F**).

#### 3.2.4 Skin rash

**Supplementary Table S15** summarizes the univariate logistic analysis results for various indices and the occurrence of skin rash as a single adverse event. Due to multicollinearity among SMA, FFM, and SMI (VIF > 10), SMI was considered more clinically meaningful and was thus selected for the multivariate logistic regression analysis. Based on variables with P < 0.05, SMI and drug dose were chosen for the multivariate regression analysis.

Similarly, the LASSO regression analysis identified three variables with non-zero coefficients: SMI, drug dose, and C-KIT13 (**Supplementary Figure S3**). However, due to the small sample size for C-KIT13, there is a risk of model fitting bias. Consequently, the final multivariate analysis included SMI (P=0.172) and drug dose (P=0.876) as the variables (**Supplementary Table S16**). Both variables had P-values > 0.05, indicating a weak relationship between these variables and the occurrence of skin rash.

#### 3.2.5 Granulocytopenia

**Supplementary Table S17** displays the results of the univariate logistic analysis for various indices related to the occurrence of granulocytopenia as a single adverse event. Variables with P-values < 0.05 were subsequently included in the multivariate regression analysis (**Supplementary Table S18**), which revealed that SMI and serum albumin had significant P-values (<0.05). The LASSO regression analysis also identified SMI and drug dose as significant variables with non-zero lambda coefficients (**Supplementary Figures S4A, B**). Two models were constructed for granulocytopenia incidence: one included SMI (P=0.011) and serum albumin (P<0.001) (**Supplementary Table S19**), while the other included SMI (P=0.673) and drug dose (P=0.076) (**Supplementary Table S20**). In the first model, both SMI and serum albumin had P-values < 0.05, indicating their significant impact on the occurrence of granulocytopenia.

To further illustrate the predictive model for a single adverse event, granulocytopenia, we developed a nomogram (**Supplementary Figure S4D**). This model achieved an AUC value of 0.793 (**Supplementary Figure S4C**) and demonstrated good calibration (**Supplementary Figure S4E**). The decision curve analysis indicated that the model has good clinical utility for predicting granulocytopenia incidence (**Supplementary Figure S4F**).

### 3.3 Relationship between actual plasma concentration of imatinib and adverse events

Out of the 107 patients analyzed, 25 underwent plasma concentration testing for imatinib at the First Affiliated Hospital of Nanjing Medical University. These tests measured the trough concentrations of imatinib. To determine the actual plasma concentration per tablet, the measured values were divided by the actual daily dose of imatinib (number of tablets) taken by the patients.

**Supplementary Table S21** presents the results of the univariate logistic analysis, examining the relationship between the actual plasma concentration per tablet and the occurrence of various adverse events. This analysis revealed a statistically significant association between the actual plasma concentration per tablet and the incidence of skin rash (P=0.046). Following this finding, we performed both univariate and multivariate logistic regression analyses specifically for skin rash (**Supplementary Table S22**). The multivariate analysis identified LAMA/SMA% (OR=1.185, P=0.031) and actual plasma concentration (OR=1.006, P=0.036) as significant risk factors for the occurrence of skin rash.

### 3.4 Factors associated with DFS

**Figure 5:**
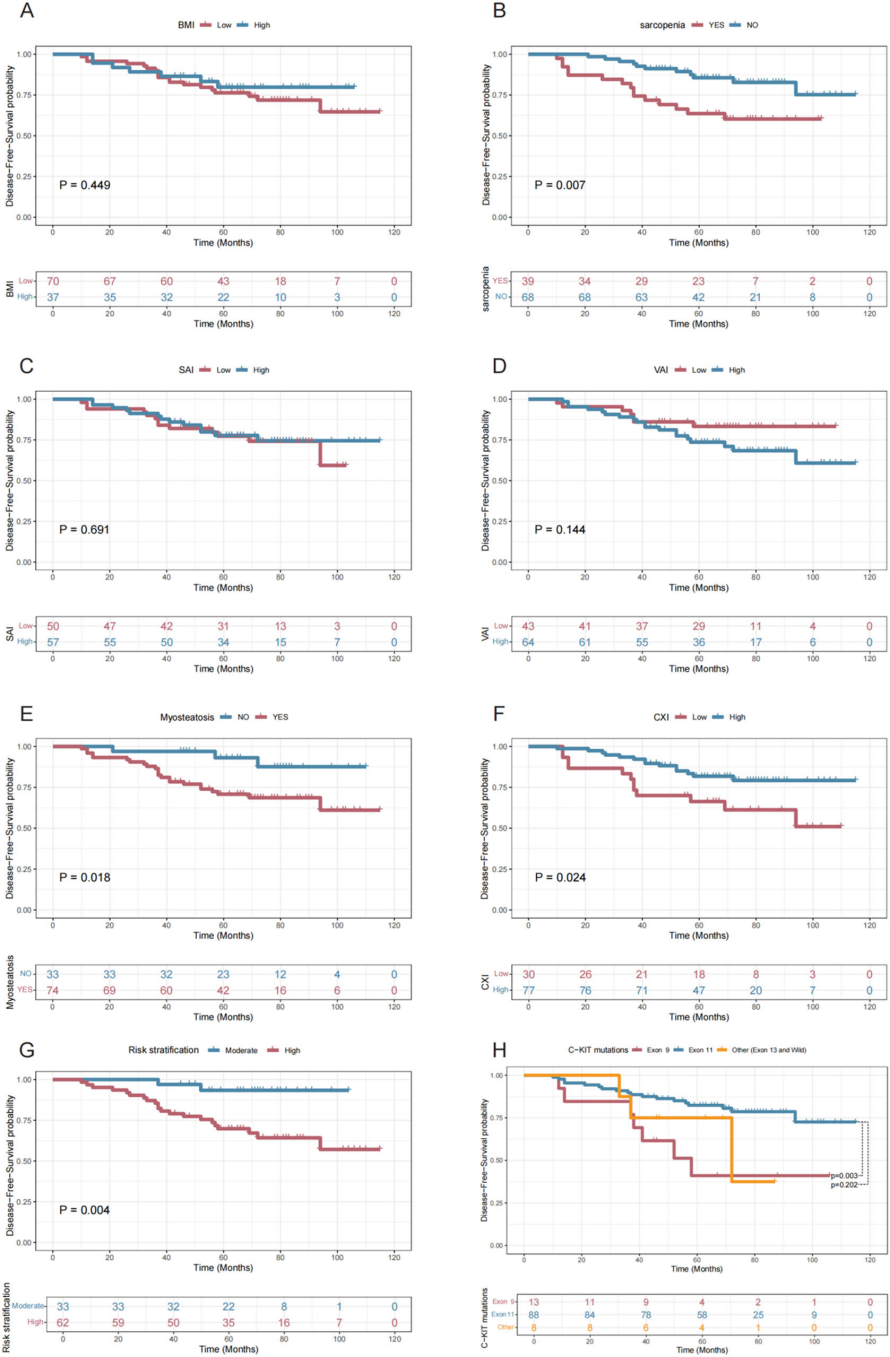
Disease-Free Survival Curves Stratified by Various Factors. This figure presents Kaplan-Meier survival curves showing disease-free survival probabilities over time for different patient groups stratified by BMI **(A)**, Sarcopenia **(B)**, SAI **(C)**, VAI **(D)**, Myosteatosis **(E)**, CXI **(F)**, Risk Stratification **(G)**, and C-KIT Mutation Types **(H)**, highlighting significant differences in outcomes for Sarcopenia (P = 0.007), Myosteatosis (P = 0.018), CXI (P = 0.024), Risk Stratification (P = 0.004), and Exon 9 vs. Exon 11 mutations (P = 0.003).

At the final follow-up in July 2024, 26 out of 107 patients (24.30%) experienced disease recurrence, with a median follow-up period of 67 months. **Figure 5** illustrates the relationships between various indices and disease-free survival. Comparative analysis of DFS among all patients revealed that those with sarcopenia (P=0.007, **Figure 5B**), myosteatosis (P=0.018, **Figure 5E**), and lower CXI (P=0.024, **Figure 5F**) had significantly poorer prognoses. Additionally, high-risk patients (P=0.004 vs. moderate-risk, **Figure 5G**) and those with C-KIT exon 9 mutation (P=0.003 vs. C-KIT exon 11 mutation, **Figure 5H**) also exhibited worse survival outcomes. BMI group (P=0.449, **Figure 5A**), SAI group (P=0.691, **Figure 5C**), VAI group (P=0.144, **Figure 5D**) had no statistical significance on DFS.

**Supplementary Table S23** summarizes the results of the univariate Cox regression analysis for various indices and Disease-free survival. The optimal cut-off values for SMI, VAI, SAI, and CXI were determined using the Youden index method, as detailed in the Materials and Methods section. The univariate analysis identified several significant predictors of DFS, including sarcopenia (P=0.010), LAMA/SMA% (P=0.024), myosteatosis (P=0.027), CXI group (P=0.029), risk stratification (P=0.013), and C-KIT mutations (P=0.011). Further multivariate regression analysis (**Supplementary Table S24**) revealed that CXI group (P=0.296) was not significant and was subsequently excluded from the final multivariate Cox regression analysis (**Table 2**). The independent risk factors for DFS were identified as sarcopenia (HR=3.067 vs. no sarcopenia, P=0.013), myosteatosis (HR=6.985 vs. no myosteatosis, P=0.024), high-risk classification (HR=9.562 vs. moderate-risk, P=0.003), and C-KIT exon 9 mutation (HR=3.615 vs. C-KIT exon 11 mutation, P=0.013).

**Table 2:** Disease-free survival multivariate COX analysis.

Variables	p-value	HR	95% CI lower limit	95% CI upper limit
Sarcopenia				
NO	0.013	0.326	0.135	0.790
YES	0.013	3.067	1.266	7.428
Myosteatosis				
NO	0.024	0.143	0.026	0.775
YES	0.024	6.985	1.291	37.796
Risk stratification				
Moderate ^a^	1.000			
High	0.003	9.562	2.126	43.007
Unknown	0.109	5.119	0.693	37.803
C-KIT mutations				
Exon 11^a^	1.000			
Exon 9	0.013	3.615	1.318	9.916
Exon 13	0.082	8.307	0.765	90.239
Exon 9 + 11	0.022	21.080	1.548	286.978
Exon 11 + 13	0.017	23.070	1.753	303.612
Wild	0.751	0.719	0.094	5.510

HR, Hazard Ratio; CI, Confidence Interval.

a Reference categories.

**Figure 6:**
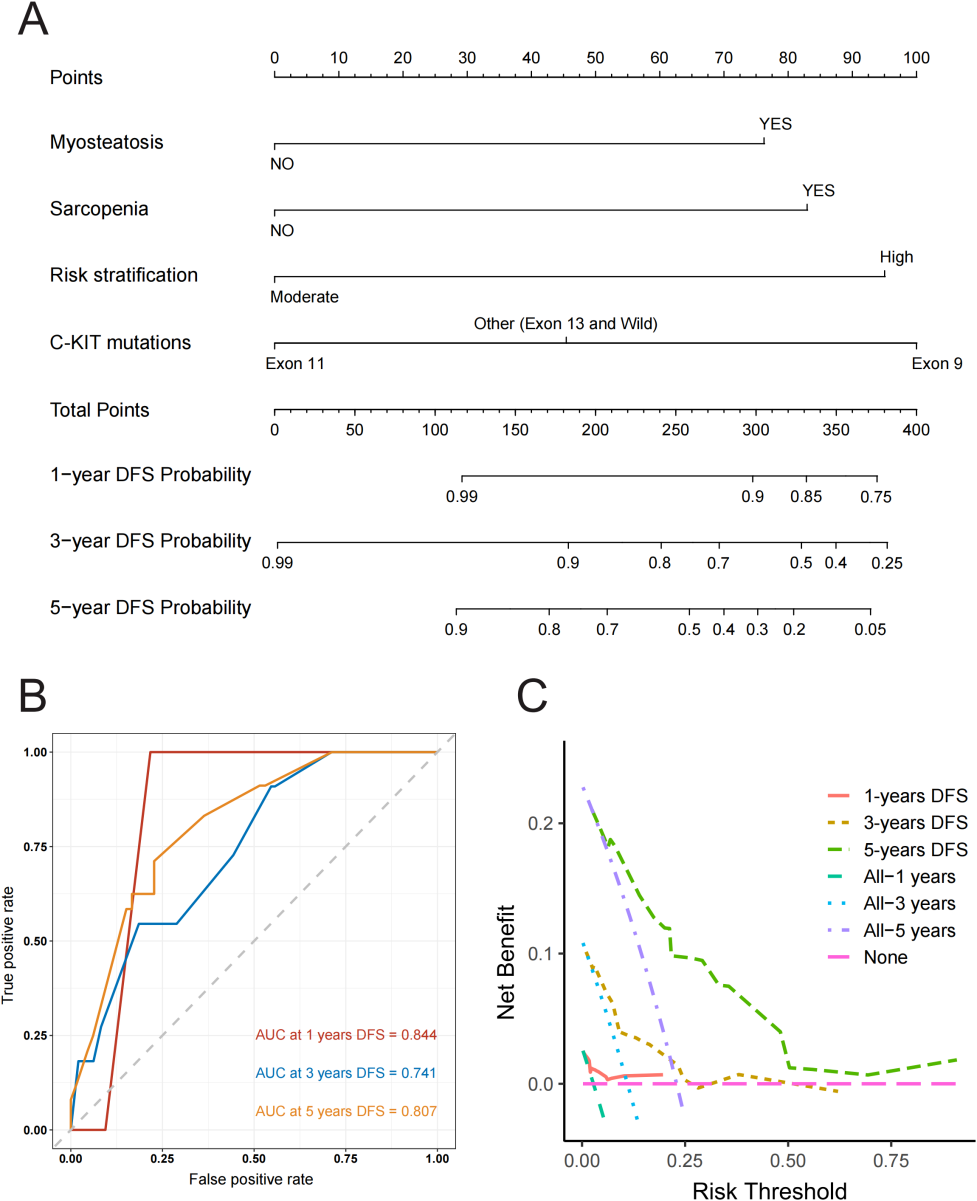
**(A)** Nomogram for predicting disease-free survival at 1, 3, and 5 years with myosteatosis status, sarcopenia status, risk stratification and C-KIT mutations. Note: The probability of each variable was added to converted into total score, and a vertical line was drawn on the total score to achieve the related probability of recurrence. **(B)** ROC Curves for Predicting Disease-Free Survival at 1, 3, and 5 Years. This figure shows ROC curves with AUC values of 0.844, 0.741, and 0.807 for predicting 1, 3, and 5-year disease-free survival respectively, illustrating the model’s accuracy in distinguishing between patients with and without recurrence at each time point. **(C)** Decision Curve Analysis (DCA) for Predicting Disease-Free Survival at 1, 3, and 5 Years. This figure presents the Decision Curve Analysis demonstrating the net benefit of the predictive model for 1, 3, and 5-year disease-free survival compared to treating all or no patients, illustrating its clinical utility in guiding treatment decisions across different risk thresholds.

Based on the final multivariate Cox regression model, we developed a nomogram (**Figure 6A**) to predict the probability of 1-year, 3-year, and 5-year DFS. The ROC curves for the nomogram demonstrated strong predictive performance, with an AUC of 0.844 for 1-year DFS, 0.741 for 3-year DFS, and 0.807 for 5-year DFS (**Figure 6B**). Calibration curves at different time points closely aligned with the standard 45-degree line, indicating high accuracy between predicted and actual DFS values (**Supplementary Figure S5**). Furthermore, the decision curve analysis curve indicated that the nomogram had good clinical utility for predicting DFS in GIST patients treated with imatinib (**Figure 6C**). Overall, our model effectively predicts DFS by integrating these critical assessment parameters.

## 4 Discussion

In this study, we evaluated the impact of baseline body composition metrics on the occurrence of treatment-related adverse events and disease-free survival in 107 GIST patients treated with imatinib. Our analysis identified myosteatosis, drug dose, and lower CXI as independent risk factors for adverse events. Additionally, LAMA/SMA% was found to be an independent risk factor for dose-limiting toxicity. These findings underscore the importance of a comprehensive assessment of muscle quality in predicting treatment-related adverse events. This knowledge can assist in identifying patients at higher risk of toxicity and in developing tailored strategies to minimize treatment interruptions.

Although the univariate analysis showed a significant association between SMI and the occurrence of any adverse events and dose-limiting toxicity, SMI was not identified as a significant factor in the multivariate analysis. Currently, no studies have established a relationship between SMI and imatinib-related toxicity. However, other research has shown that low SMI is a significant predictor of dose-limiting toxicity in sorafenib treatment for renal cell carcinoma (30). *Hong S*’s study found that both low SMI and myosteatosis predicted early chemotherapy-related toxicity in pancreatic cancer patients (19). In contrast, our study found that SMI was not a predictor of adverse events or dose-limiting toxicity. Instead, myosteatosis, which indicates fat infiltration in muscles, was a significant predictor of toxicity although there is currently no universal definition of sarcopenia. In our analysis, we treated SMI as a continuous variable rather than categorizing it as high or low. When SMI and myosteatosis were included in the multivariate analysis together, the increased degrees of freedom caused SMI to lose statistical significance. Despite this, we believe that while myosteatosis is distinct from sarcopenia, there is a close relationship between the two, and myosteatosis may be a better predictor of imatinib-related drug toxicity than SMI.

Cancer cachexia is a complex, multi-organ catabolic syndrome that severely impacts patients’ quality of life and increases their susceptibility to adverse reactions from chemotherapy (31, 32). Our study also demonstrated that a lower CXI is an independent risk factor for adverse events and edema. Although CXI was not included in the multivariate analysis for disease-free survival due to a P-value > 0.05, the impact of CXI on the incidence of imatinib-related adverse events cannot be overlooked.

*Yi Qian* et al. (33) demonstrated that adverse events such as edema, leukopenia, and rash are significantly associated with imatinib plasma concentrations in GIST patients. In our analysis of 25 patients with available plasma concentration data, we also found that higher plasma concentrations of imatinib correlate with more severe skin rash adverse events. However, due to technical challenges and patient compliance issues, conducting trough plasma concentration tests for every patient is impractical. Therefore, we evaluated the drug dose by calculating the total drug concentration per FFM. Our results indicated that higher drug doses are independent risk factors for adverse events and granulocytopenia.

Considering the significant role of these indicators in predicting imatinib-related adverse events, we developed predictive nomograms for the incidence of both overall and single adverse events. These quantitative models exhibit high goodness of fit and provide clinical benefits. Prior to this, no studies had quantitatively predicted imatinib toxicity. Clinically, these nomograms allow for increased monitoring frequency for patients with a high predicted probability of adverse events. This enables enhanced surveillance for potential toxicities, preventive medication use, and appropriate supportive care.

In our analysis of disease-free survival with imatinib, the final predictive factors included sarcopenia, myosteatosis, risk stratification, and C-KIT mutations. Risk stratification is based on tumor size, mitotic rate (specify the number of mitoses per 5mm²), and tumor site, with high-risk patients having a higher probability of recurrence (23). The response of GIST to imatinib varies with the primary KIT mutation genotype; patients with C-KIT exon 9 or 13 mutations have shorter median recurrence times on imatinib compared to those with C-KIT exon 11 mutations (34). In *Song H’*s study, sarcopenia, surgical resection method, and mitotic index were included in a nomogram predicting overall survival in GIST patients (35). Our nomogram for predicting DFS in GIST patients treated with imatinib incorporates all significant indicators from previous studies and introduces myosteatosis as a new predictive factor. This nomogram demonstrates reliable performance, with an AUC value of 0.807 for predicting 5-year DFS. Decision curve analysis also shows that the nomogram provides high clinical benefit across a wide range of threshold probabilities for predicting 5-year DFS.

Given the poor prognoses and higher rates of adverse events in GIST patients with myosteatosis and sarcopenia, improving these conditions is crucial. Current treatments for these obesity-related diseases focus on lifestyle interventions, including dietary control and physical exercise. Research indicates that supplementing diets with essential amino acids and branched-chain amino acids can improve sarcopenia (36). Fructose intake, which promotes oxidative stress and mitochondrial dysfunction, leads to fat accumulation in muscle and increased autophagy in muscle cells. Consequently, a low added-sugar diet can help prevent myosteatosis (37). Vitamin D is essential for maintaining muscle tissue homeostasis (38). Additionally, antioxidants such as selenium, carotenoids, tocopherols, flavonoids, and polyphenols support protein synthesis and reduce protein breakdown, thereby improving sarcopenia (39). Alongside dietary changes, physical activity is vital. Exercise enhances skeletal muscle mass, physical function, and muscle strength, potentially reversing sarcopenia and myosteatosis (36). Therefore, a home-based physical activity program, based on a mix of aerobic and resistance mild-intensity exercises, is essential for patients with these conditions. Recent studies have identified mitochondrial uncouplers like BAM15 and SHC517, and Glycine and N-Acetylcysteine (GlyNAC) supplementation, as promising treatments for sarcopenic obesity (40). For GIST patients on imatinib, these cost-effective strategies to improve myosteatosis can reduce treatment-related adverse events and dose-limiting toxicity, while also prolonging disease-free survival. This highlights the significant clinical value of our study.

This study has several limitations. First, it is a single-center retrospective study with a small sample size. Due to limited clinical data and the low incidence of GIST, only 107 patients were recruited, leading to unavoidable selection bias. The applicability of the results to other patient groups requires further internal and external validation. Despite these limitations, this study remains one of the largest reports on imatinib-related adverse events and survival outcomes in GIST. Second, there is no international consensus on the definitions of various indicators, including myosteatosis and sarcopenia. Myosteatosis refers to the abnormal accumulation of fat cells in muscle tissue, but a unified standard for its definition has not yet been established. We adopted the criteria from *Hong S*’s study (19). According to the European Working Group on Sarcopenia in Older People (EWGSOP), sarcopenia should be assessed using parameters such as muscle mass, muscle strength, and physical performance (41). Due to the retrospective nature of this study, we defined sarcopenia solely based on muscle mass. Prospective studies incorporating additional sarcopenia assessment tools are needed. Finally, because GIST patients generally have long overall survival times, we only analyzed DFS with imatinib. We will continue to collect long-term survival data for these patients to further validate the impact of myosteatosis and sarcopenia on survival outcomes.

Given the significance of our findings for GIST patients, we also plan to further investigate the molecular biological associations of myosteatosis through metabolomics sequencing of tumor and hematological specimens. This new study is already underway, and updated findings will be available in a future version of this study.

## 5 Conclusion

In summary, our findings suggest that baseline CT-detected myosteatosis, drug dose, and the CXI index can effectively predict treatment-related toxicity in GIST patients undergoing imatinib therapy. By carefully selecting treatment plans and dosages, and providing additional medical support for patients with baseline myosteatosis, we can minimize treatment interruptions and improve patient compliance. Additionally, we conducted a comprehensive analysis of the relationship between preoperative sarcopenia, myosteatosis, and survival outcomes, resulting in the development of a new nomogram to accurately predict 1-year, 3-year, and 5-year disease-free survival in GIST patients. Therefore, this study provides valuable insights that can help physicians make better clinical assessments and treatment decisions.

## Data Availability

The original contributions presented in the study are included in the article/**Supplementary Material**. Further inquiries can be directed to the corresponding author/s.
