# Supplementary material for: Using body composition to predict treatment-related adverse events and disease-free survival in patients with gastrointestinal stromal tumors treated with imatinib: a retrospective cohort study
Source: Front Immunol. 2025 Apr 7;16:1576834. doi: 10.3389/fimmu.2025.1576834 (PMC12009723; doi:10.3389/fimmu.2025.1576834)
Supplement: Supplementary file 1 [file DataSheet1.docx]

**Supplementary Fig. S1**


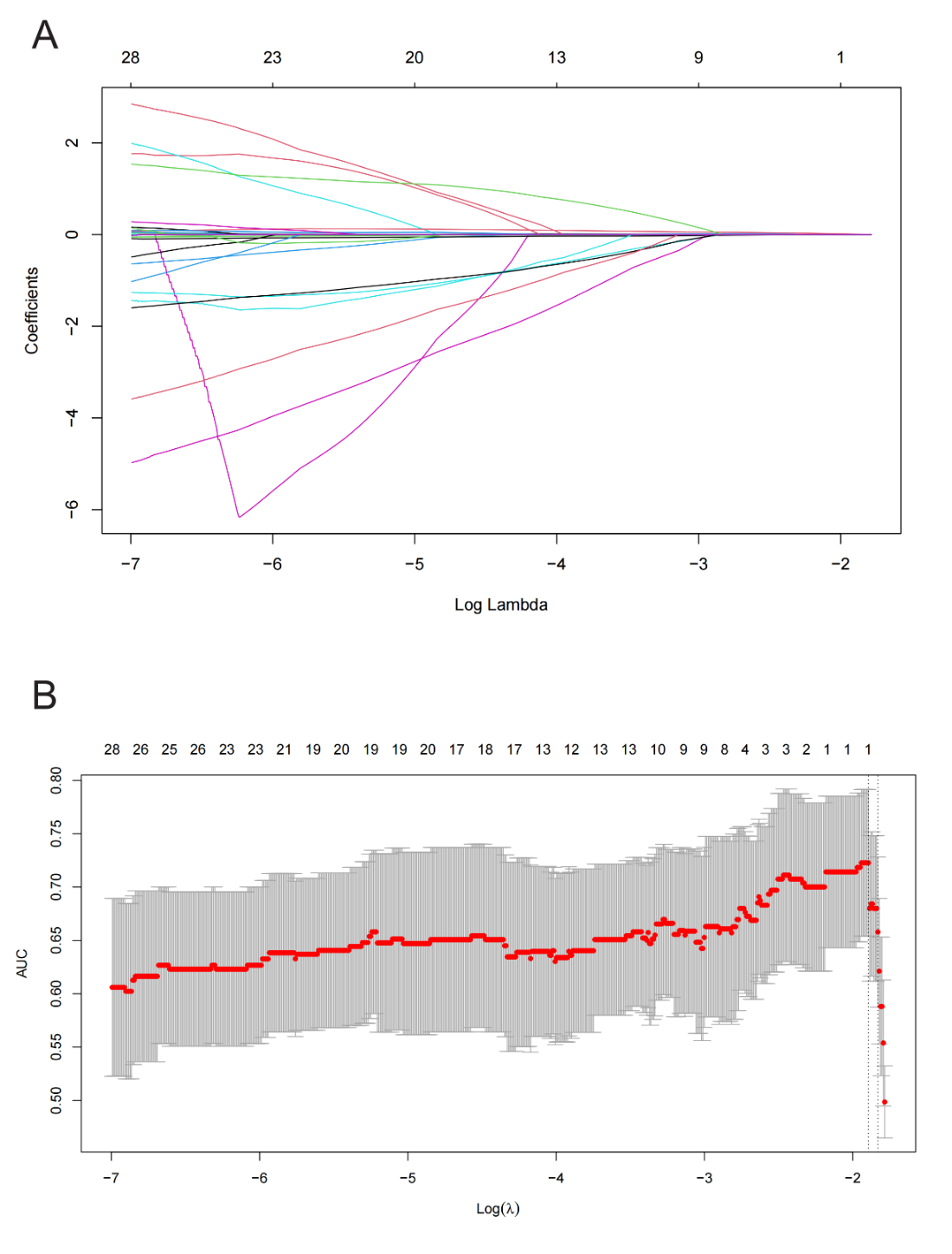


**(A)** LASSO Regression Coefficient Path. This figure shows the trajectories of regression coefficients for various predictors of dose-limiting toxicity as a function of the logarithm of the regularization parameter (Log Lambda) in a LASSO regression model, illustrating the process of variable selection and regularization. **(B)** AUC vs. Log Lambda in LASSO Regression. This figure illustrates the relationship between the Area Under the Curve (AUC) and the logarithm of the regularization parameter (Log Lambda) in a LASSO regression model, showing how AUC changes with varying regularization and indicating the number of predictors of dose-limiting toxicity that remain at each value of Log Lambda.

**Supplementary Fig. S2**


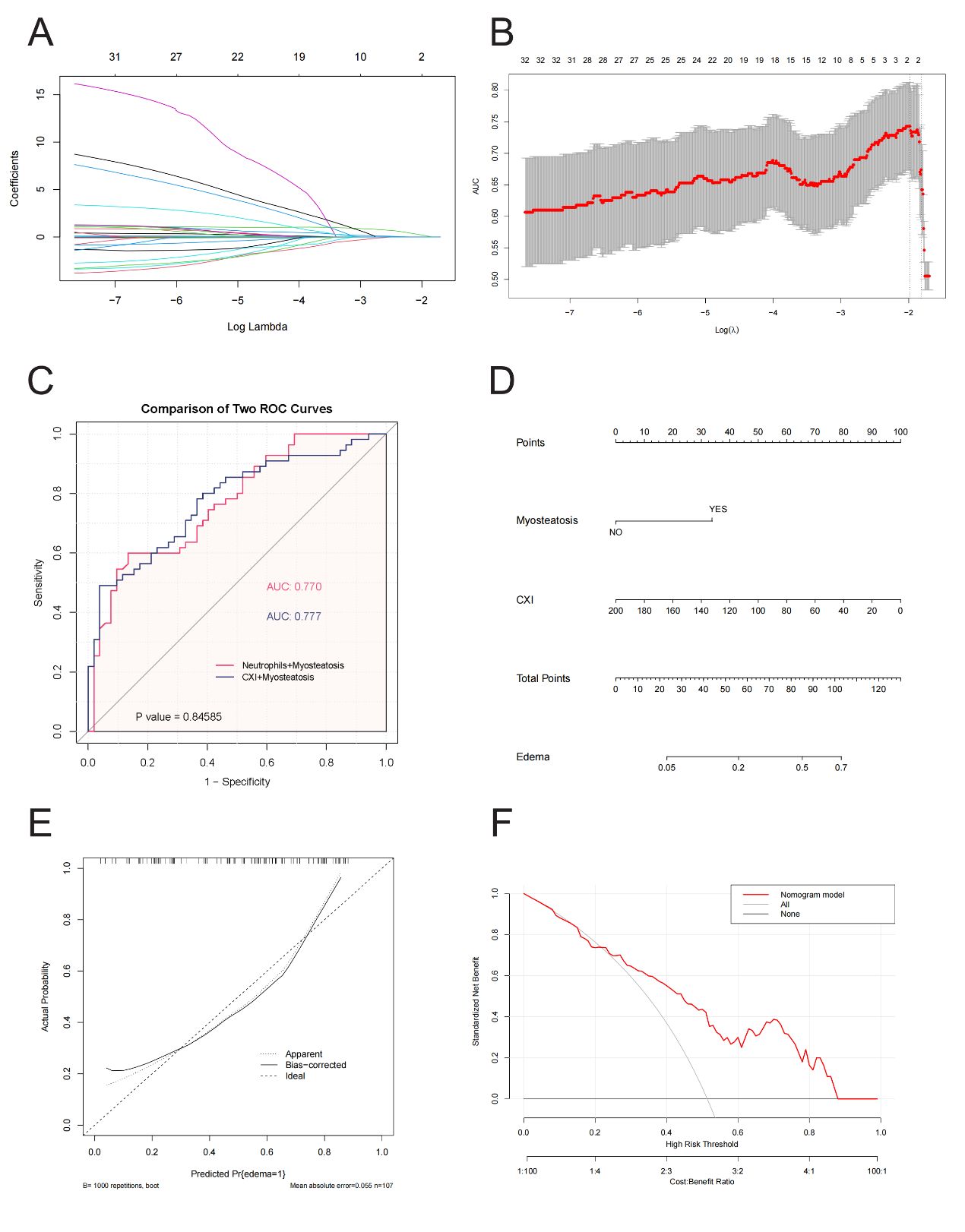


**(A)** LASSO Regression Coefficient Path. This figure shows the trajectories of regression coefficients for various predictors of edema as the logarithm of the regularization parameter (Log Lambda) changes in a LASSO regression model, illustrating the process of variable selection and regularization. **(B)** AUC vs. Log Lambda in LASSO Regression. This figure shows the relationship between the Area Under the Curve (AUC) and the logarithm of the regularization parameter (Log Lambda) in a LASSO regression model, illustrating how AUC values change with different levels of regularization and indicating the number of predictors of edema that remain at each Log Lambda value. **(C)** Comparison of Two ROC Curves for Predicting Edema. This figure compares the ROC curves of two predictive models, Neutrophils + Myosteatosis (AUC = 0.770) and CXI + Myosteatosis (AUC = 0.777), showing similar predictive accuracy with a P value of 0.84585, indicating no significant difference between the models. **(D)** Nomogram for Predicting the Probability of Edema. This nomogram predicts the probability of edema based on points assigned for the presence of myosteatosis and CXI, with total points correlating to edema probability, providing a visual tool for clinicians to estimate patient risk. **(E)** Calibration Curve for Predicting the Probability of Edema. This calibration curve shows the predicted versus actual probabilities of edema, with the apparent, bias-corrected (using 1000 bootstrap repetitions), and ideal lines illustrating the model's accuracy, indicated by a mean absolute error of 0.055 based on 107 patients. **(F)** Decision Curve Analysis (DCA) for Predicting Edema. This Decision Curve Analysis (DCA) compares the net benefit of using the nomogram model for predicting edema against the strategies of treating all patients and treating none, across various risk thresholds and cost-benefit ratios.

**Supplementary Fig. S3**


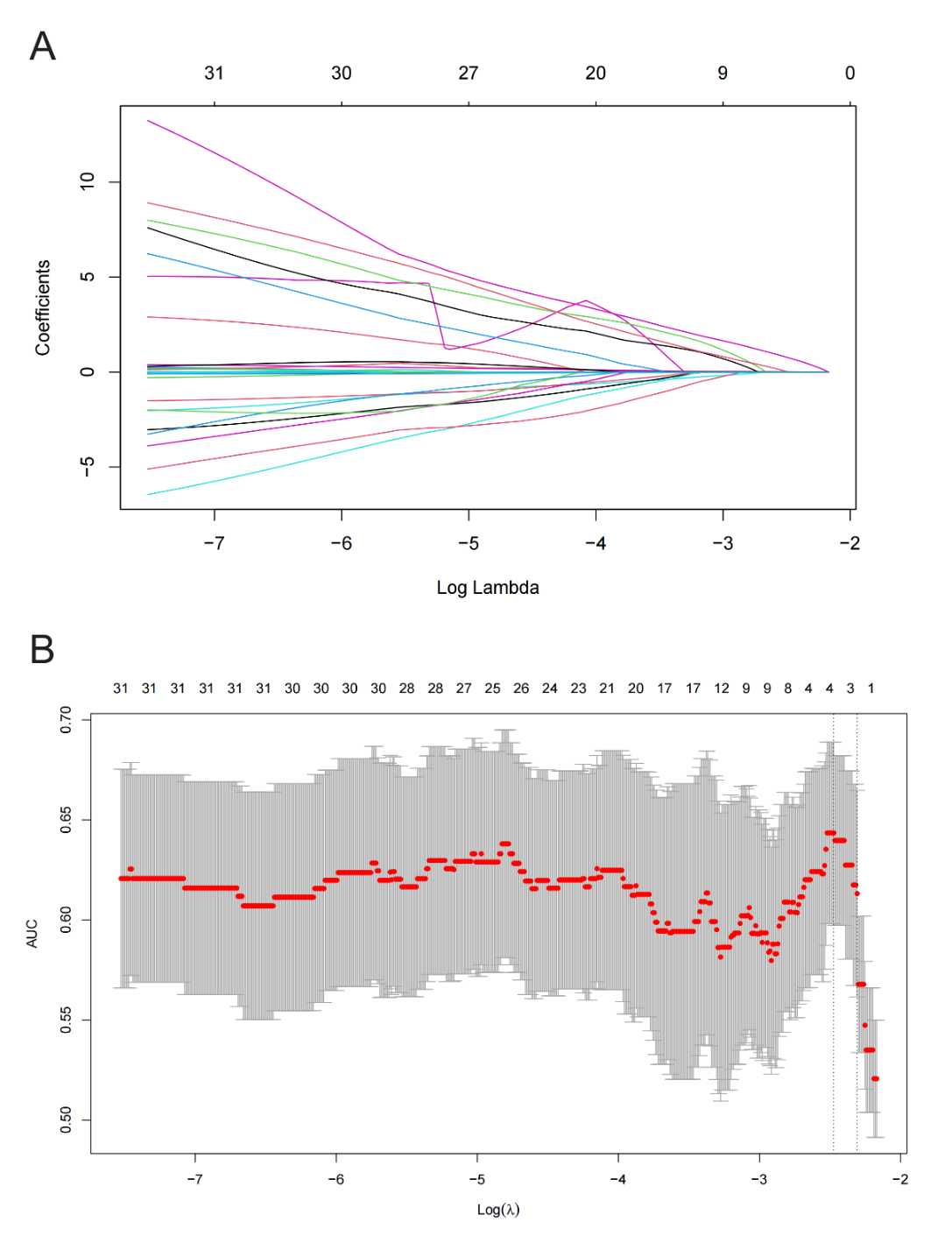


**(A)** LASSO Regression Coefficient Path. This figure illustrates the trajectories of regression coefficients for various predictors of skin rash as the logarithm of the regularization parameter (Log Lambda) changes, showing the variable selection and regularization process in a LASSO regression model. **(B)** AUC vs. Log Lambda in LASSO Regression. This figure illustrates the relationship between AUC and the logarithm of the regularization parameter (Log Lambda) in a LASSO regression model, showing how AUC values change with different levels of regularization and indicating the number of predictors of skin rash that remain at each Log Lambda value.

**Supplementary Fig. S4**


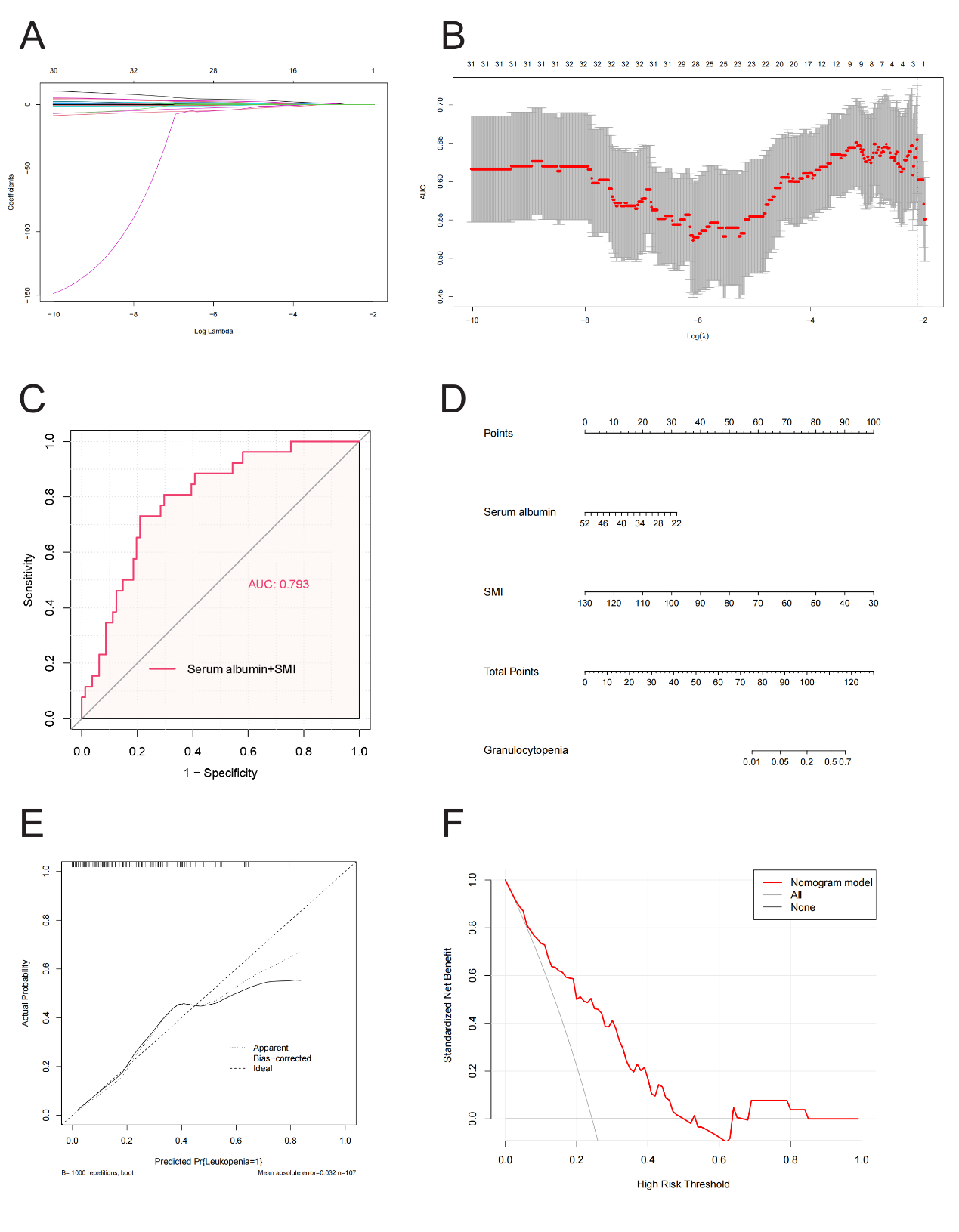


**(A)** LASSO Regression Coefficient Path. This figure illustrates the trajectories of regression coefficients for various predictors of granulocytopenia as the logarithm of the regularization parameter (Log Lambda) changes, demonstrating the variable selection and regularization process in a LASSO regression model. **(B)** AUC vs. Log Lambda in LASSO Regression. This figure illustrates the relationship between AUC and the logarithm of the regularization parameter (Log Lambda) in a LASSO regression model, showing how AUC values change with different levels of regularization and indicating the number of predictors of granulocytopenia that remain at each Log Lambda value. **(C)** ROC Curve for Predicting Granulocytopenia. This figure shows the ROC curve with an AUC of 0.793, indicating good predictive accuracy for a model using Serum Albumin and Skeletal Muscle Index (SMI) to predict granulocytopenia. **(D)** Nomogram for Predicting the Probability of Granulocytopenia. This nomogram predicts the probability of granulocytopenia based on Serum Albumin levels and Skeletal Muscle Index (SMI), with total points correlating to the granulocytopenia probability, providing a visual tool for clinicians to estimate patient risk. **(E)** Calibration Curve for Predicting the Probability of Granulocytopenia. This calibration curve shows the predicted versus actual probabilities of granulocytopenia, with the apparent, bias-corrected (using 1000 bootstrap repetitions), and ideal lines illustrating the model's accuracy, indicated by a mean absolute error of 0.032 based on 107 patients. **(F)** Decision Curve Analysis (DCA) for Predicting Granulocytopenia. This Decision Curve Analysis (DCA) shows the net benefit of using the nomogram model for predicting granulocytopenia compared to treating all patients or none, demonstrating its potential clinical value in guiding treatment decisions across different risk thresholds.

**Supplementary Fig. S5**


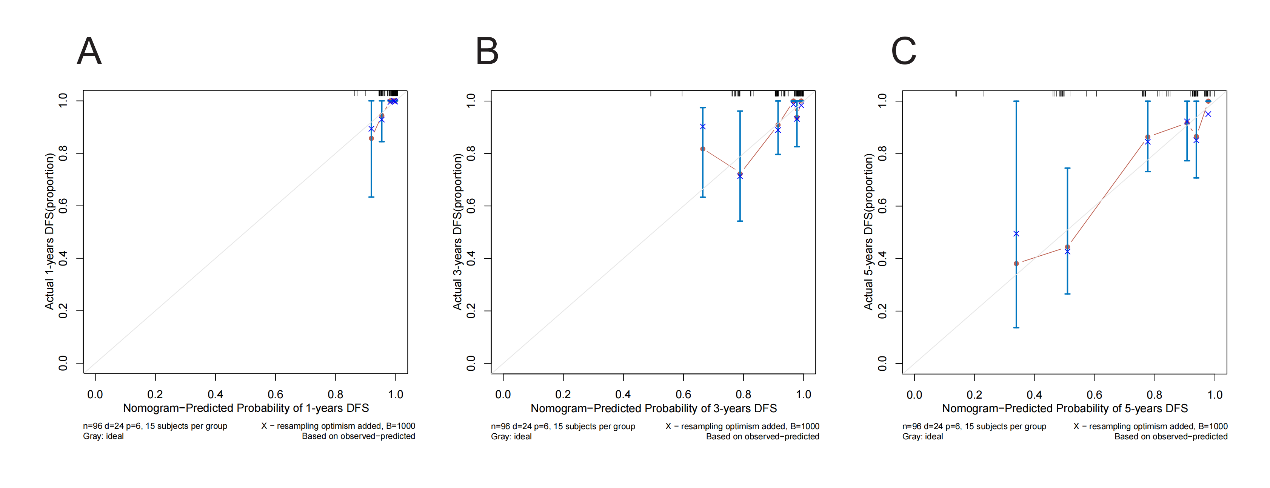


Calibration Curves for 1, 3, and 5-Year Disease-Free Survival (DFS). Calibration curves of the prognostic nomogram for 1-years disease-free survival **(A)**, 3-years disease-free survival **(B)**, 5-years disease-free survival **(C)**. The Y-axis indicates the observed disease-free survival of GIST while the X-axis indicates the estimated disease-free survival. The solid line demonstrates the ideal reference line that predicted GIST survival associated with the actual outcome whereas the dashed line demonstrates the prediction of nomogram. The closer alignment with the solid line represents the better performance is acquired.
