# Supplementary material for: Using body composition to predict treatment-related adverse events and disease-free survival in patients with gastrointestinal stromal tumors treated with imatinib: a retrospective cohort study
Source: Front Immunol. 2025 Apr 7;16:1576834. doi: 10.3389/fimmu.2025.1576834 (PMC12009723; doi:10.3389/fimmu.2025.1576834)
Supplement: Supplementary file 2 [file Table1.docx]

**Supplementary Table S1.**

Adverse Events Univariate Logistic Analysis

| **Variables** | **p-value** | **OR** | **95% CI lower limit** | **95% CI upper limit** |
| --- | --- | --- | --- | --- |
| Age | 0.494 | 1.014 | 0.974 | 1.055 |
| Sex |  |  |  |  |
| Female | 0.001 | 5.600 | 2.073 | 17.993 |
| Male | 0.001 | 0.179 | 0.056 | 0.482 |
| Height | 0.117 | 0.008 | 0.000 | 2.966 |
| Weight | 0.065 | 0.958 | 0.914 | 1.001 |
| BMI | 0.242 | 0.918 | 0.792 | 1.057 |
| Serum albumin | 0.134 | 0.935 | 0.852 | 1.018 |
| Neutrophils | 0.563 | 1.078 | 0.842 | 1.414 |
| Lymphocyte | 0.062 | 0.494 | 0.227 | 1.023 |
| SMA | 0.016 | 0.982 | 0.966 | 0.995 |
| SAA | 0.397 | 1.004 | 0.996 | 1.013 |
| VAA | 0.040 | 0.993 | 0.985 | 1.000 |
| LAMA | 0.102 | 1.033 | 0.997 | 1.079 |
| SMI | 0.019 | 0.941 | 0.891 | 0.984 |
| SAI | 0.243 | 1.013 | 0.992 | 1.038 |
| VAI | 0.082 | 0.982 | 0.962 | 1.002 |
| LAMA/SMA % | 0.001 | 1.109 | 1.049 | 1.183 |
| Myosteatosis |  |  |  |  |
| NO | 0.000 | 0.115 | 0.042 | 0.293 |
| YES | 0.000 | 8.686 | 3.417 | 23.563 |
| CXI | 0.011 | 0.985 | 0.974 | 0.996 |
| FFM | 0.016 | 0.941 | 0.892 | 0.984 |
| Drug dose | 0.001 | 1.456 | 1.198 | 1.841 |
| Tumor site |  |  |  |  |
| Gastric ^a^ | 1.000 |  |  |  |
| Duodenum | 0.644 | 0.732 | 0.202 | 3.040 |
| Jejunum/ileum | 0.307 | 0.585 | 0.209 | 1.662 |
| Colon | 0.995 | 4580886.000 | 0.000 | NA |
| Rectum | 0.397 | 0.293 | 0.011 | 7.770 |
| Abdominal | 0.892 | 1.171 | 0.154 | 24.056 |
| Pelvic | 0.995 | 4580886.000 | 0.000 | NA |
| Liver | 0.995 | 4580886.000 | 0.000 | NA |
| Unknown | 0.130 | 0.146 | 0.006 | 1.653 |
| Risk stratification |  |  |  |  |
| Moderate ^a^ | 1.000 |  |  |  |
| High | 0.329 | 0.609 | 0.226 | 1.646 |
| Unknown | 0.787 | 0.808 | 0.171 | 3.807 |
| C-KIT mutations |  |  |  |  |
| Exon 9 ^a^ | 1.000 |  |  |  |
| Exon 11 | 0.633 | 1.370 | 0.340 | 4.798 |
| Exon 13 | 0.991 | 7825680.000 | 0.000 | NA |
| Exon 9+11 | 0.995 | 7825680.000 | 0.000 | NA |
| Exon 11+13 | 0.994 | 0.000 | NA | 4.07188E+204 |
| Wild | 0.756 | 1.500 | 0.134 | 35.899 |
| PDGFR mutations |  |  |  |  |
| Exon 18 ^a^ | 1.000 |  |  |  |
| Wild | 0.991 | 0.000 | NA | 3.929181E+121 |

Abbreviations: BMI, Body Mass Index; SMA, Skeletal Muscle Area; SAA, Subcutaneous Adipose Area; VAA, Visceral Adipose Area; LAMA, Low Attenuation Muscle Area; SMI, Skeletal Muscle Index; SAI, Subcutaneous Adipose Index; VAI, Visceral Adipose Index; CXI, Cachexia Index; FFM, Fat-Free Mass; OR, Odds Ratio; CI, Confidence Interval

^a^ Reference categories

**Supplementary Table S2.**

Adverse Events Multivariate Logistic Analysis

| **Variables** | **p-value** | **OR** | **95% CI lower limit** | **95% CI upper limit** |
| --- | --- | --- | --- | --- |
| Sex(Male) | 0.681 | 0.735 | 0.170 | 3.186 |
| SMI | 0.485 | 1.020 | 0.964 | 1.080 |
| Myosteatosis(YES) | 0.001 | 7.245 | 2.356 | 22.282 |
| CXI | 0.014 | 0.982 | 0.968 | 0.996 |
| Drug dose | 0.048 | 1.417 | 1.004 | 2.000 |

Abbreviations: SMI, Skeletal Muscle Index; CXI, Cachexia Index; OR, Odds Ratio; CI, Confidence Interval

Hosmer-Lemeshow test : P-value=0.880

**Supplementary Table S3.**

Adverse Events Multivariate Logistic Analysis

| **Variables** | **p-value** | **OR** | **95% CI lower limit** | **95% CI upper limit** |
| --- | --- | --- | --- | --- |
| Sex(Male) | 0.261 | 0.449 | 0.111 | 1.816 |
| SMI | 0.596 | 1.015 | 0.962 | 1.070 |
| LAMA/SMA % | 0.034 | 1.075 | 1.006 | 1.149 |
| CXI | 0.023 | 0.985 | 0.972 | 0.998 |
| Drug dose | 0.137 | 1.287 | 0.923 | 1.796 |

Abbreviations: SMI, Skeletal Muscle Index; CXI, Cachexia Index; OR, Odds Ratio; CI, Confidence Interval

Hosmer-Lemeshow test : P-value=0.040

**Supplementary Table S4.**

Adverse Events Multivariate Logistic Analysis

| **Variables** | **p-value** | **OR** | **95% CI lower limit** | **95% CI upper limit** |
| --- | --- | --- | --- | --- |
| Myosteatosis(YES) | <0.001 | 7.640 | 2.596 | 22.480 |
| CXI | 0.017 | 0.983 | 0.969 | 0.997 |
| Drug dose | 0.010 | 1.349 | 1.076 | 1.693 |

Abbreviations: CXI, Cachexia Index; OR, Odds Ratio; CI, Confidence Interval

Hosmer-Lemeshow test : P-value=0.356

**Supplementary Table S5.**

Adverse Events Multivariate Logistic Analysis

| **Variables** | **p-value** | **OR** | **95% CI lower limit** | **95% CI upper limit** |
| --- | --- | --- | --- | --- |
| VAA | 0.026 | 0.989 | 0.980 | 0.999 |
| Myosteatosis(YES) | <0.001 | 12.204 | 3.465 | 42.979 |
| CXI | 0.018 | 0.982 | 0.967 | 0.997 |
| Drug dose | 0.042 | 1.285 | 1.009 | 1.636 |

Abbreviations: VAA, Visceral Adipose Area; CXI, Cachexia Index; OR, Odds Ratio; CI, Confidence Interval

Hosmer-Lemeshow test : P-value=0.036

**Supplementary Table S6.**

DLT Univariate Logistic Analysis

| **Variables** | **p-value** | **OR** | **95% CI lower limit** | **95% CI upper limit** |
| --- | --- | --- | --- | --- |
| Age | 0.011 | 1.063 | 1.014 | 1.115 |
| Sex |  |  |  |  |
| Female | 0.067 | 2.299 | 0.944 | 5.597 |
| Male | 0.067 | 0.435 | 0.179 | 1.059 |
| Height | 0.020 | 0.001 | 0.000 | 0.309 |
| Weight | 0.015 | 0.944 | 0.901 | 0.989 |
| BMI | 0.172 | 0.904 | 0.782 | 1.045 |
| Serum albumin | 0.153 | 0.940 | 0.865 | 1.023 |
| Neutrophils | 0.755 | 0.960 | 0.742 | 1.242 |
| Lymphocyte | 0.246 | 0.614 | 0.269 | 1.401 |
| SMA | 0.007 | 0.976 | 0.959 | 0.993 |
| SAA | 0.671 | 0.998 | 0.990 | 1.007 |
| VAA | 0.981 | 1.000 | 0.993 | 1.007 |
| LAMA | 0.153 | 1.022 | 0.992 | 1.054 |
| SMI | 0.021 | 0.935 | 0.883 | 0.990 |
| SAI | 0.996 | 1.000 | 0.979 | 1.021 |
| VAI | 0.630 | 1.005 | 0.985 | 1.025 |
| LAMA/SMA % | 0.000 | 1.095 | 1.043 | 1.150 |
| Myosteatosis |  |  |  |  |
| NO | 0.017 | 0.208 | 0.058 | 0.751 |
| YES | 0.017 | 4.800 | 1.331 | 17.311 |
| CXI | 0.034 | 0.986 | 0.973 | 0.999 |
| FFM | 0.007 | 0.923 | 0.871 | 0.978 |
| Drug dose | 0.003 | 1.288 | 1.091 | 1.521 |
| Tumor site |  |  |  |  |
| Gastric ^a^ | 1.000 |  |  |  |
| Duodenum | 0.078 | 0.150 | 0.018 | 1.236 |
| Jejunum/ileum | 0.282 | 0.556 | 0.190 | 1.621 |
| Colon | 1.000 | 0.000 | 0.000 | NA |
| Rectum | 0.645 | 1.944 | 0.115 | 32.933 |
| Abdominal | 0.532 | 0.486 | 0.051 | 4.676 |
| Pelvic | 1.000 | 0.000 | 0.000 | NA |
| Liver | 1.000 | 0.000 | 0.000 | NA |
| Unknown | 0.999 | 0.000 | 0.000 | NA |
| Risk stratification |  |  |  |  |
| Moderate ^a^ | 1.000 |  |  |  |
| High | 0.038 | 0.369 | 0.144 | 0.946 |
| Unknown | 0.167 | 0.308 | 0.058 | 1.636 |
| C-KIT mutations |  |  |  |  |
| Exon 9 ^a^ | 1.000 |  |  |  |
| Exon 11 | 0.459 | 1.825 | 0.372 | 8.965 |
| Exon 13 | 0.527 | 2.500 | 0.146 | 42.800 |
| Exon 9+11 | 1.000 | 0.000 | 0.000 | NA |
| Exon 11+13 | 1.000 | 0.000 | 0.000 | NA |
| Wild | 0.713 | 1.667 | 0.109 | 25.433 |
| PDGFR mutations |  |  |  |  |
| Exon 18 ^a^ | 1.000 |  |  |  |
| Wild | 1.000 | 0.000 | 0.000 | NA |

Abbreviations: DLT, Dose-Limiting Toxicity; BMI, Body Mass Index; SMA, Skeletal Muscle Area; SAA, Subcutaneous Adipose Area; VAA, Visceral Adipose Area; LAMA, Low Attenuation Muscle Area; SMI, Skeletal Muscle Index; SAI, Subcutaneous Adipose Index; VAI, Visceral Adipose Index; CXI, Cachexia Index; FFM, Fat-Free Mass; OR, Odds Ratio; CI, Confidence Interval

^a^ Reference categories

**Supplementary Table S7.**

DLT Multivariate Logistic Analysis

| **Variables** | **p-value** | **OR** | **95% CI lower limit** | **95% CI upper limit** |
| --- | --- | --- | --- | --- |
| Age | 0.055 | 1.054 | 0.999 | 1.111 |
| Height | 0.961 | 1.260 | 0.000 | 12109.442 |
| Weight | 0.193 | 0.957 | 0.895 | 1.023 |
| SMI | 0.797 | 1.008 | 0.947 | 1.074 |
| Myosteatosis(YES) | 0.157 | 2.700 | 0.682 | 10.692 |
| CXI | 0.108 | 0.989 | 0.975 | 1.002 |
| Drug dose | 0.399 | 1.133 | 0.848 | 1.514 |

Abbreviations: DLT, Dose-Limiting Toxicity; SMI, Skeletal Muscle Index; CXI, Cachexia Index; OR, Odds Ratio; CI, Confidence Interval

Hosmer-Lemeshow test : P-value=0.965

**Supplementary Table S8.**

DLT Multivariate Logistic Analysis

| **Variables** | **p-value** | **OR** | **95% CI lower limit** | **95% CI upper limit** |
| --- | --- | --- | --- | --- |
| Age | 0.276 | 1.033 | 0.975 | 1.095 |
| Height | 0.956 | 0.769 | 0.000 | 8346.101 |
| Weight | 0.105 | 0.944 | 0.880 | 1.012 |
| SMI | 0.926 | 1.003 | 0.938 | 1.074 |
| LAMA/SMA % | 0.028 | 1.072 | 1.007 | 1.141 |
| CXI | 0.161 | 0.990 | 0.976 | 1.004 |
| Drug dose | 0.831 | 1.034 | 0.759 | 1.409 |

Abbreviations: DLT, Dose-Limiting Toxicity; SMI, Skeletal Muscle Index; CXI, Cachexia Index; OR, Odds Ratio; CI, Confidence Interval

Hosmer-Lemeshow test : P-value=0.687

**Supplementary Table S9.**

Edema Univariate Logistic Analysis

| **Variables** | **p-value** | **OR** | **95% CI lower limit** | **95% CI upper limit** |
| --- | --- | --- | --- | --- |
| Age | 0.462 | 1.013 | 0.978 | 1.050 |
| Sex |  |  |  |  |
| Female | 0.024 | 2.471 | 1.126 | 5.421 |
| Male | 0.024 | 0.405 | 0.184 | 0.888 |
| Height | 0.719 | 0.385 | 0.002 | 70.365 |
| Weight | 0.573 | 0.989 | 0.952 | 1.027 |
| BMI | 0.584 | 0.966 | 0.853 | 1.094 |
| Serum albumin | 0.016 | 0.905 | 0.834 | 0.981 |
| Neutrophils | 0.004 | 1.470 | 1.129 | 1.915 |
| Lymphocyte | 0.086 | 0.541 | 0.268 | 1.090 |
| SMA | 0.405 | 0.995 | 0.985 | 1.006 |
| SAA | 0.615 | 1.002 | 0.995 | 1.009 |
| VAA | 0.241 | 0.996 | 0.990 | 1.003 |
| LAMA | 0.022 | 1.040 | 1.006 | 1.076 |
| SMI | 0.369 | 0.984 | 0.950 | 1.019 |
| SAI | 0.610 | 1.005 | 0.987 | 1.023 |
| VAI | 0.246 | 0.989 | 0.972 | 1.007 |
| LAMA/SMA % | 0.003 | 1.067 | 1.022 | 1.114 |
| Myosteatosis |  |  |  |  |
| NO | 0.001 | 0.228 | 0.093 | 0.561 |
| YES | 0.001 | 4.381 | 1.784 | 10.761 |
| CXI | 0.000 | 0.978 | 0.965 | 0.990 |
| FFM | 0.405 | 0.985 | 0.950 | 1.021 |
| Drug dose | 0.048 | 1.154 | 1.001 | 1.329 |
| Tumor site |  |  |  |  |
| Gastric ^a^ | 1.000 |  |  |  |
| Duodenum | 0.950 | 1.038 | 0.320 | 3.373 |
| Jejunum/ileum | 0.813 | 1.118 | 0.442 | 2.827 |
| Colon | 1.000 | 1677608490.653 | 0.000 | NA |
| Rectum | 0.979 | 1.038 | 0.062 | 17.486 |
| Abdominal | 0.642 | 1.558 | 0.240 | 10.091 |
| Pelvic | 1.000 | 1677608490.653 | 0.000 | NA |
| Liver | 1.000 | 1677608490.653 | 0.000 | NA |
| Unknown | 0.602 | 0.519 | 0.044 | 6.078 |
| Risk stratification |  |  |  |  |
| Moderate ^a^ | 1.000 |  |  |  |
| High | 0.888 | 1.063 | 0.456 | 2.473 |
| Unknown | 0.285 | 2.125 | 0.534 | 8.453 |
| C-KIT mutations |  |  |  |  |
| Exon 9 ^a^ | 1.000 |  |  |  |
| Exon 11 | 0.642 | 0.748 | 0.220 | 2.542 |
| Exon 13 | 0.448 | 0.357 | 0.025 | 5.109 |
| Exon 9+11 | 1.000 | 1153910602.037 | 0.000 | NA |
| Exon 11+13 | 1.000 | 0.000 | 0.000 | NA |
| Wild | 0.772 | 0.714 | 0.074 | 6.922 |
| PDGFR mutations |  |  |  |  |
| Exon 18 ^a^ | 1.000 |  |  |  |
| Wild | 1.000 | 0.000 | 0.000 | NA |

Abbreviations: BMI, Body Mass Index; SMA, Skeletal Muscle Area; SAA, Subcutaneous Adipose Area; VAA, Visceral Adipose Area; LAMA, Low Attenuation Muscle Area; SMI, Skeletal Muscle Index; SAI, Subcutaneous Adipose Index; VAI, Visceral Adipose Index; CXI, Cachexia Index; FFM, Fat-Free Mass; OR, Odds Ratio; CI, Confidence Interval

^a^ Reference categories

**Supplementary Table S10.**

Edema Multivariate Logistic Analysis

| **Variables** | **p-value** | **OR** | **95% CI lower limit** | **95% CI upper limit** |
| --- | --- | --- | --- | --- |
| Sex(Male) | 0.161 | 0.391 | 0.105 | 1.452 |
| Serum albumin | 0.168 | 0.925 | 0.827 | 1.034 |
| Neutrophils | 0.028 | 1.493 | 1.043 | 2.136 |
| Myosteatosis(YES) | 0.005 | 4.779 | 1.596 | 14.307 |
| CXI | 0.240 | 0.990 | 0.973 | 1.007 |
| Drug dose | 0.956 | 1.006 | 0.804 | 1.260 |

Abbreviations: CXI, Cachexia Index; OR, Odds Ratio; CI, Confidence Interval

Hosmer-Lemeshow test : P-value=0.631

**Supplementary Table S11.**

Edema Multivariate Logistic Analysis

| **Variables** | **p-value** | **OR** | **95% CI lower limit** | **95% CI upper limit** |
| --- | --- | --- | --- | --- |
| Sex(Male) | 0.065 | 0.300 | 0.084 | 1.077 |
| Serum albumin | 0.234 | 0.937 | 0.841 | 1.043 |
| Neutrophils | 0.030 | 1.451 | 1.037 | 2.030 |
| LAMA/SMA % | 0.031 | 1.059 | 1.005 | 1.116 |
| CXI | 0.255 | 0.990 | 0.974 | 1.007 |
| Drug dose | 0.671 | 0.952 | 0.758 | 1.195 |

Abbreviations: LAMA, Low Attenuation Muscle Area; SMA, Skeletal Muscle Area; CXI, Cachexia Index; OR, Odds Ratio; CI, Confidence Interval

Hosmer-Lemeshow test : P-value=0.601

**Supplementary Table S12.**

Edema Multivariate Logistic Analysis

| **Variables** | **p-value** | **OR** | **95% CI lower limit** | **95% CI upper limit** |
| --- | --- | --- | --- | --- |
| Neutrophils | 0.001 | 1.639 | 1.228 | 2.189 |
| Myosteatosis(YES) | <0.001 | 6.772 | 2.398 | 19.125 |

Abbreviations: OR, Odds Ratio; CI, Confidence Interval

Hosmer-Lemeshow test : P-value=0.134

**Supplementary Table S13.**

Edema Multivariate Logistic Analysis

| **Variables** | **p-value** | **OR** | **95% CI lower limit** | **95% CI upper limit** |
| --- | --- | --- | --- | --- |
| Neutrophils | 0.001 | 1.568 | 1.193 | 2.06 |
| LAMA/SMA % | 0.001 | 1.084 | 1.033 | 1.138 |

Abbreviations: LAMA, Low Attenuation Muscle Area; SMA, Skeletal Muscle Area; OR, Odds Ratio; CI, Confidence Interval

Hosmer-Lemeshow test : P-value=0.05

**Supplementary Table S14.**

Edema Multivariate Logistic Analysis

| **Variables** | **p-value** | **OR** | **95% CI lower limit** | **95% CI upper limit** |
| --- | --- | --- | --- | --- |
| CXI | <0.001 | 0.976 | 0.964 | 0.990 |
| Myosteatosis(YES) | 0.001 | 4.989 | 1.883 | 13.214 |

Abbreviations: CXI, Cachexia Index; OR, Odds Ratio; CI, Confidence Interval

Hosmer-Lemeshow test : P-value=0.185

**Supplementary Table S15.**

Skin Rash Univariate Logistic Analysis

| **Variables** | **p-value** | **OR** | **95% CI lower limit** | **95% CI upper limit** |
| --- | --- | --- | --- | --- |
| Age | 0.853 | 0.996 | 0.960 | 1.034 |
| Sex |  |  |  |  |
| Female | 0.500 | 1.322 | 0.587 | 2.977 |
| Male | 0.500 | 0.756 | 0.336 | 1.703 |
| Height | 0.843 | 1.753 | 0.007 | 448.673 |
| Weight | 0.285 | 0.978 | 0.939 | 1.019 |
| BMI | 0.211 | 0.918 | 0.803 | 1.050 |
| Serum albumin | 0.871 | 0.994 | 0.919 | 1.074 |
| Neutrophils | 0.274 | 0.870 | 0.678 | 1.117 |
| Lymphocyte | 0.148 | 0.567 | 0.263 | 1.223 |
| SMA | 0.038 | 0.985 | 0.970 | 0.999 |
| SAA | 0.564 | 1.002 | 0.995 | 1.010 |
| VAA | 0.481 | 0.998 | 0.991 | 1.004 |
| LAMA | 0.694 | 0.994 | 0.964 | 1.025 |
| SMI | 0.013 | 0.936 | 0.889 | 0.986 |
| SAI | 0.594 | 1.005 | 0.986 | 1.024 |
| VAI | 0.521 | 0.994 | 0.975 | 1.013 |
| LAMA/SMA % | 0.248 | 1.023 | 0.984 | 1.064 |
| Myosteatosis |  |  |  |  |
| NO | 0.216 | 0.557 | 0.221 | 1.406 |
| YES | 0.216 | 1.795 | 0.711 | 4.533 |
| CXI | 0.499 | 0.996 | 0.986 | 1.007 |
| FFM | 0.038 | 0.949 | 0.904 | 0.997 |
| Drug dose | 0.024 | 1.187 | 1.023 | 1.378 |
| Tumor site |  |  |  |  |
| Gastric ^a^ | 1.000 |  |  |  |
| Duodenum | 0.216 | 0.416 | 0.103 | 1.669 |
| Jejunum/ileum | 0.228 | 0.533 | 0.192 | 1.482 |
| Colon | 1.000 | 2461675951.011 | 0.000 | NA |
| Rectum | 0.770 | 1.524 | 0.090 | 25.717 |
| Abdominal | 0.402 | 0.381 | 0.040 | 3.648 |
| Pelvic | 1.000 | 0.000 | 0.000 | NA |
| Liver | 1.000 | 2461675951.011 | 0.000 | NA |
| Unknown | 0.999 | 0.000 | 0.000 | NA |
| Risk stratification |  |  |  |  |
| Moderate ^a^ | 1.000 |  |  |  |
| High | 0.391 | 0.680 | 0.281 | 1.643 |
| Unknown | 0.377 | 0.513 | 0.117 | 2.256 |
| C-KIT mutations |  |  |  |  |
| Exon 9 ^a^ | 1.000 |  |  |  |
| Exon 11 | 0.339 | 2.167 | 0.443 | 10.587 |
| Exon 13 | 0.999 | 8077374214.256 | 0.000 | NA |
| Exon 9+11 | 1.000 | 8077374214.256 | 0.000 | NA |
| Exon 11+13 | 1.000 | 0.000 | 0.000 | NA |
| Wild | 0.051 | 15.000 | 0.983 | 228.896 |
| PDGFR mutations |  |  |  |  |
| Exon 18 ^a^ | 1.000 |  |  |  |
| Wild | 1.000 | 0.000 | 0.000 | NA |

Abbreviations: BMI, Body Mass Index; SMA, Skeletal Muscle Area; SAA, Subcutaneous Adipose Area; VAA, Visceral Adipose Area; LAMA, Low Attenuation Muscle Area; SMI, Skeletal Muscle Index; SAI, Subcutaneous Adipose Index; VAI, Visceral Adipose Index; CXI, Cachexia Index; FFM, Fat-Free Mass; OR, Odds Ratio; CI, Confidence Interval

^a^ Reference categories

**Supplementary Table S16.**

Skin Rash Multivariate Logistic Analysis

| **Variables** | **p-value** | **OR** | **95% CI lower limit** | **95% CI upper limit** |
| --- | --- | --- | --- | --- |
| SMI | 0.172 | 0.941 | 0.863 | 1.027 |
| Drug dose | 0.876 | 1.020 | 0.793 | 1.313 |

Abbreviations: SMI, Skeletal Muscle Index; OR, Odds Ratio; CI, Confidence Interval

Hosmer-Lemeshow test : P-value=0.360

**Supplementary Table S17.**

Granulocytopenia Univariate Logistic Analysis

| **Variables** | **p-value** | **OR** | **95% CI lower limit** | **95% CI upper limit** |
| --- | --- | --- | --- | --- |
| Age | 0.646 | 1.010 | 0.969 | 1.053 |
| Sex |  |  |  |  |
| Female | 0.013 | 3.211 | 1.273 | 8.100 |
| Male | 0.013 | 0.311 | 0.123 | 0.786 |
| Height | 0.011 | 0.000 | 0.000 | 0.140 |
| Weight | 0.006 | 0.934 | 0.890 | 0.981 |
| BMI | 0.099 | 0.882 | 0.761 | 1.024 |
| Serum albumin | 0.028 | 0.906 | 0.830 | 0.990 |
| Neutrophils | 0.377 | 1.118 | 0.873 | 1.431 |
| Lymphocyte | 0.349 | 1.426 | 0.679 | 2.994 |
| SMA | 0.000 | 0.963 | 0.944 | 0.983 |
| SAA | 0.954 | 1.000 | 0.992 | 1.008 |
| VAA | 0.054 | 0.992 | 0.983 | 1.000 |
| LAMA | 0.796 | 0.996 | 0.963 | 1.029 |
| SMI | 0.001 | 0.893 | 0.836 | 0.954 |
| SAI | 0.543 | 1.006 | 0.986 | 1.027 |
| VAI | 0.135 | 0.983 | 0.962 | 1.005 |
| LAMA/SMA % | 0.014 | 1.057 | 1.011 | 1.104 |
| Myosteatosis |  |  |  |  |
| NO | 0.147 | 0.451 | 0.153 | 1.324 |
| YES | 0.147 | 2.219 | 0.755 | 6.517 |
| CXI | 0.164 | 0.991 | 0.979 | 1.004 |
| FFM | 0.000 | 0.883 | 0.825 | 0.945 |
| Drug dose | 0.001 | 1.325 | 1.116 | 1.573 |
| Tumor site |  |  |  |  |
| Gastric ^a^ | 1.000 |  |  |  |
| Duodenum | 0.420 | 0.513 | 0.101 | 2.598 |
| Jejunum/ileum | 0.891 | 1.077 | 0.372 | 3.121 |
| Colon | 1.000 | 0.000 | 0.000 | NA |
| Rectum | 0.438 | 3.077 | 0.179 | 52.746 |
| Abdominal | 0.458 | 2.051 | 0.308 | 13.652 |
| Pelvic | 1.000 | 0.000 | 0.000 | NA |
| Liver | 1.000 | 4970691824.157 | 0.000 | NA |
| Unknown | 0.999 | 0.000 | 0.000 | NA |
| Risk stratification |  |  |  |  |
| Moderate ^a^ | 1.000 |  |  |  |
| High | 0.239 | 1.948 | 0.643 | 5.902 |
| Unknown | 0.068 | 4.000 | 0.901 | 17.763 |
| C-KIT mutations |  |  |  |  |
| Exon 9 ^a^ | 1.000 |  |  |  |
| Exon 11 | 0.263 | 3.333 | 0.405 | 27.422 |
| Exon 13 | 0.055 | 22.000 | 0.938 | 515.872 |
| Exon 9+11 | 1.000 | 17770223271.363 | 0.000 | NA |
| Exon 11+13 | 1.000 | 0.000 | 0.000 | NA |
| Wild | 0.097 | 11.000 | 0.646 | 187.166 |
| PDGFR mutations |  |  |  |  |
| Exon 18 ^a^ | 1.000 |  |  |  |
| Wild | 1.000 | 0.000 | 0.000 | NA |

Abbreviations: BMI, Body Mass Index; SMA, Skeletal Muscle Area; SAA, Subcutaneous Adipose Area; VAA, Visceral Adipose Area; LAMA, Low Attenuation Muscle Area; SMI, Skeletal Muscle Index; SAI, Subcutaneous Adipose Index; VAI, Visceral Adipose Index; CXI, Cachexia Index; FFM, Fat-Free Mass; OR, Odds Ratio; CI, Confidence Interval

^a^ Reference categories

**Supplementary Table S18.**

Granulocytopenia Multivariate Logistic Analysis

| **Variables** | **p-value** | **OR** | **95% CI lower limit** | **95% CI upper limit** |
| --- | --- | --- | --- | --- |
| Sex(Male) | 0.285 | 3.181 | 0.381 | 26.571 |
| Height | 0.086 | 0.000 | 0.000 | 5.759 |
| Weight | 0.978 | 1.001 | 0.930 | 1.078 |
| Serum albumin | 0.023 | 0.874 | 0.779 | 0.981 |
| SMI | 0.021 | 0.855 | 0.747 | 0.977 |
| LAMA/SMA % | 0.401 | 1.024 | 0.969 | 1.081 |
| Drug dose | 0.625 | 0.924 | 0.673 | 1.268 |

Abbreviations: SMI, Skeletal Muscle Index; LAMA, Low Attenuation Muscle Area; SMA, Skeletal Muscle Area; OR, Odds Ratio; CI, Confidence Interval

Hosmer-Lemeshow test : P-value=0.342

**Supplementary Table S19.**

Granulocytopenia Multivariate Logistic Analysis

| **Variables** | **p-value** | **OR** | **95% CI lower limit** | **95% CI upper limit** |
| --- | --- | --- | --- | --- |
| Serum albumin | 0.011 | 0.872 | 0.784 | 0.970 |
| SMI | 0.000 | 0.878 | 0.817 | 0.944 |

Abbreviations: SMI, Skeletal Muscle Index; OR, Odds Ratio; CI, Confidence Interval

Hosmer-Lemeshow test : P-value=0.836

**Supplementary Table S20.**

Granulocytopenia Multivariate Logistic Analysis

| **Variables** | **p-value** | **OR** | **95% CI lower limit** | **95% CI upper limit** |
| --- | --- | --- | --- | --- |
| Drug dose | 0.673 | 1.062 | 0.803 | 1.404 |
| SMI | 0.076 | 0.909 | 0.818 | 1.010 |

Abbreviations: SMI, Skeletal Muscle Index; OR, Odds Ratio; CI, Confidence Interval

Hosmer-Lemeshow test : P-value=0.834

**Supplementary Table S21.**

Plasma Concentration Univariate Logistic Analysis

| **Variables** | **p-value** | **OR** | **95% CI lower limit** | **95% CI upper limit** |
| --- | --- | --- | --- | --- |
| Adverse events | 0.925 | 1.000 | 0.993 | 1.006 |
| Dose-Limiting Toxicity | 0.880 | 1.000 | 0.995 | 1.004 |
| Edema | 0.188 | 0.997 | 0.992 | 1.002 |
| Skin rash | 0.046 | 1.006 | 1.000 | 1.011 |
| Granulocytopenia | 0.547 | 0.999 | 0.994 | 1.003 |
| Anemia | 0.913 | 1.000 | 0.994 | 1.007 |
| Thrombocytopenia | 0.551 | 0.997 | 0.988 | 1.007 |
| Nausea and vomiting | 0.318 | 0.997 | 0.991 | 1.003 |
| Diarrhea | 0.535 | 0.998 | 0.992 | 1.004 |
| Dyspepsia | 0.533 | 0.910 | 0.676 | 1.225 |
| Liver dysfunction | 0.922 | 1.000 | 0.992 | 1.008 |

Abbreviations: OR, Odds Ratio; CI, Confidence Interval

**Supplementary Table S22.**

Univariate and Multivariable Logistic Analyses of Skin Rash

|  | **Skin Rash Univariate Analysis** | | | |  | **Skin Rash Multivariate Analysis** | | | |
| --- | --- | --- | --- | --- | --- | --- | --- | --- | --- |
| **Variables** | **p-value** | **OR** | **95% CI lower limit** | **95% CI upper limit** |  | **p-value** | **OR** | **95% CI lower limit** | **95% CI upper limit** |
| Age | 0.324 | 1.038 | 0.963 | 1.119 |  | - |  |  |  |
| Sex |  |  |  |  |  |  |  |  |  |
| Female | 0.423 | 0.500 | 0.092 | 2.730 |  | - |  |  |  |
| Male | 0.423 | 2.000 | 0.366 | 10.919 |  | - |  |  |  |
| Height | 0.512 | 52.318 | 0.000 | 7176747.684 |  | - |  |  |  |
| Weight | 0.497 | 0.974 | 0.904 | 1.050 |  | - |  |  |  |
| BMI | 0.199 | 0.805 | 0.578 | 1.121 |  | - |  |  |  |
| Serum albumin | 0.110 | 0.795 | 0.601 | 1.053 |  | - |  |  |  |
| Neutrophils | 0.708 | 0.925 | 0.614 | 1.392 |  | - |  |  |  |
| Lymphocyte | 0.059 | 0.207 | 0.040 | 1.063 |  | - |  |  |  |
| SMA | 0.475 | 0.989 | 0.958 | 1.020 |  | - |  |  |  |
| SAA | 0.858 | 1.002 | 0.983 | 1.021 |  | - |  |  |  |
| VAA | 0.481 | 0.995 | 0.981 | 1.009 |  | - |  |  |  |
| LAMA | 0.062 | 1.093 | 0.996 | 1.199 |  | - |  |  |  |
| SMI | 0.283 | 0.938 | 0.835 | 1.054 |  | - |  |  |  |
| SAI | 0.925 | 0.997 | 0.946 | 1.052 |  | - |  |  |  |
| VAI | 0.428 | 0.983 | 0.943 | 1.025 |  | - |  |  |  |
| LAMA/SMA % | 0.032 | 1.156 | 1.013 | 1.321 |  | 0.031 | 1.185 | 1.015 | 1.383 |
| Myosteatosis |  |  |  |  |  |  |  |  |  |
| NO | 0.112 | 0.222 | 0.035 | 1.422 |  | - |  |  |  |
| YES | 0.112 | 4.500 | 0.703 | 28.794 |  | - |  |  |  |
| CXI | 0.171 | 0.987 | 0.968 | 1.006 |  | - |  |  |  |
| FFM | 0.266 | 0.934 | 0.828 | 1.054 |  | - |  |  |  |
| Drug dose | 0.641 | 1.093 | 0.752 | 1.588 |  | - |  |  |  |
| Plasma concentration | 0.046 | 1.006 | 1.000 | 1.011 |  | 0.036 | 1.006 | 1.000 | 1.012 |
| Tumor site |  |  |  |  |  | - |  |  |  |
| Gastric ^a^ | 1.000 |  |  |  |  |  |  |  |  |
| Jejunum/ileum | 0.795 | 0.800 | 0.149 | 4.297 |  |  |  |  |  |
| Abdominal | 1.000 | 0.000 | 0.000 | NA |  |  |  |  |  |
| Pelvic | 1.000 | 0.000 | 0.000 | NA |  |  |  |  |  |
| Risk stratification |  |  |  |  |  | - |  |  |  |
| Moderate ^a^ | 1.000 |  |  |  |  |  |  |  |  |
| High | 0.708 | 0.700 | 0.108 | 4.538 |  |  |  |  |  |
| Unknown | 1.000 | 1615474842.851 | 0.000 | NA |  |  |  |  |  |
| C-KIT mutations |  |  |  |  |  | - |  |  |  |
| Exon 11 ^a^ | 1.000 |  |  |  |  |  |  |  |  |
| Exon 9 | 1.000 | 0.000 | 0.000 | NA |  |  |  |  |  |
| Exon 13 | 1.000 | 2423212264.277 | 0.000 | NA |  |  |  |  |  |
| Wild | 0.401 | 3.000 | 0.232 | 38.875 |  |  |  |  |  |
| PDGFR |  |  |  |  |  | - |  |  |  |
| Wild ^a^ | 1.000 |  |  |  |  |  |  |  |  |

Abbreviations: BMI, Body Mass Index; SMA, Skeletal Muscle Area; SAA, Subcutaneous Adipose Area; VAA, Visceral Adipose Area; LAMA, Low Attenuation Muscle Area; SMI, Skeletal Muscle Index; SAI, Subcutaneous Adipose Index; VAI, Visceral Adipose Index; CXI, Cachexia Index; FFM, Fat-Free Mass; OR, Odds Ratio; CI, Confidence Interval

^a^ Reference categories

**Supplementary Table S23.**

Disease-Free Survival Univariate COX Analysis

| **Variables** | **p-value** | **HR** | **95% CI lower limit** | **95% CI upper limit** |
| --- | --- | --- | --- | --- |
| Age | 0.702 | 1.007 | 0.972 | 1.043 |
| Sex |  |  |  |  |
| Female | 0.542 | 1.271 | 0.589 | 2.743 |
| Male | 0.542 | 0.787 | 0.365 | 1.699 |
| Height | 0.800 | 0.509 | 0.003 | 94.020 |
| Weight | 0.412 | 0.984 | 0.948 | 1.022 |
| BMI | 0.402 | 0.947 | 0.833 | 1.076 |
| BMI group |  |  |  |  |
| <25 | 0.450 | 1.397 | 0.587 | 3.325 |
| >25 | 0.450 | 0.716 | 0.301 | 1.704 |
| Serum albumin | 0.234 | 0.957 | 0.891 | 1.029 |
| Neutrophils | 0.097 | 1.175 | 0.971 | 1.422 |
| Lymphocyte | 0.753 | 0.898 | 0.459 | 1.756 |
| SMA | 0.262 | 0.993 | 0.980 | 1.006 |
| SAA | 0.607 | 1.002 | 0.995 | 1.009 |
| VAA | 0.711 | 0.999 | 0.992 | 1.005 |
| LAMA | 0.205 | 1.015 | 0.992 | 1.039 |
| SMI | 0.209 | 0.972 | 0.929 | 1.016 |
| SAI | 0.583 | 1.005 | 0.988 | 1.023 |
| VAI | 0.717 | 0.997 | 0.978 | 1.015 |
| Sarcopenia |  |  |  |  |
| NO | 0.010 | 0.357 | 0.164 | 0.778 |
| YES | 0.010 | 2.804 | 1.286 | 6.116 |
| SAI group |  |  |  |  |
| Low | 0.691 | 1.169 | 0.541 | 2.523 |
| High | 0.691 | 0.856 | 0.396 | 1.847 |
| VAI group |  |  |  |  |
| Low | 0.151 | 0.530 | 0.223 | 1.261 |
| High | 0.151 | 1.887 | 0.793 | 4.492 |
| LAMA/SMA % | 0.024 | 1.039 | 1.005 | 1.075 |
| Myosteatosis |  |  |  |  |
| NO | 0.027 | 0.258 | 0.077 | 0.860 |
| YES | 0.027 | 3.874 | 1.163 | 12.910 |
| CXI | 0.062 | 0.989 | 0.978 | 1.001 |
| CXI group |  |  |  |  |
| Low | 0.029 | 2.373 | 1.095 | 5.143 |
| High | 0.029 | 0.421 | 0.194 | 0.914 |
| FFM | 0.262 | 0.976 | 0.934 | 1.019 |
| Drug dose | 0.212 | 1.087 | 0.953 | 1.240 |
| Tumor site |  |  |  |  |
| Gastric ^a^ | 1.000 |  |  |  |
| Duodenum | 0.320 | 2.021 | 0.505 | 8.087 |
| Jejunum/ileum | 0.002 | 4.795 | 1.796 | 12.801 |
| Colon | 0.001 | 38.970 | 4.163 | 364.691 |
| Rectum | 0.998 | 0.000 | 0.000 | Inf |
| Abdominal | 0.005 | 7.400 | 1.813 | 30.206 |
| Pelvic | 0.999 | 0.000 | 0.000 | Inf |
| Liver | 0.000 | 78.590 | 7.602 | 812.449 |
| Unknown | 0.998 | 0.000 | 0.000 | Inf |
| Risk stratification |  |  |  |  |
| Moderate ^a^ | 1.000 |  |  |  |
| High | 0.013 | 6.259 | 1.467 | 26.710 |
| Unknown | 0.092 | 4.659 | 0.778 | 27.910 |
| C-KIT mutations |  |  |  |  |
| Exon 11 ^a^ | 1.000 |  |  |  |
| Exon 9 | 0.011 | 3.415 | 1.327 | 8.789 |
| Exon 13 | 0.305 | 2.918 | 0.378 | 22.554 |
| Exon 9+11 | 0.001 | 40.260 | 4.342 | 373.340 |
| Exon 11+13 | 0.142 | 4.566 | 0.603 | 34.606 |
| Wild | 0.694 | 1.500 | 0.198 | 11.349 |
| PDGFR mutations |  |  |  |  |
| Exon 18 ^a^ | 1.000 |  |  |  |
| Wild | 0.998 | 0.000 | 0.000 | Inf |

Abbreviations: BMI, Body Mass Index; SMA, Skeletal Muscle Area; SAA, Subcutaneous Adipose Area; VAA, Visceral Adipose Area; LAMA, Low Attenuation Muscle Area; SMI, Skeletal Muscle Index; SAI, Subcutaneous Adipose Index; VAI, Visceral Adipose Index; CXI, Cachexia Index; FFM, Fat-Free Mass; HR, Hazard Ratio; CI, Confidence Interval

^a^ Reference categories

**Supplementary Table S24.**

Disease-Free Survival Multivariate COX Analysis

| **Variables** | **p-value** | **HR** | **95% CI lower limit** | **95% CI upper limit** |
| --- | --- | --- | --- | --- |
| Sarcopenia |  |  |  |  |
| NO | 0.055 | 0.395 | 0.153 | 1.018 |
| YES | 0.055 | 2.530 | 0.982 | 6.517 |
| Myosteatosis |  |  |  |  |
| NO | 0.022 | 0.149 | 0.029 | 0.761 |
| YES | 0.022 | 6.697 | 1.315 | 34.119 |
| CXI group |  |  |  |  |
| Low | 0.296 | 1.616 | 0.657 | 3.974 |
| High | 0.296 | 0.619 | 0.252 | 1.522 |
| Risk stratification |  |  |  |  |
| Moderate ^a^ | 1.000 |  |  |  |
| High | 0.006 | 8.798 | 1.893 | 40.881 |
| Unknown | 0.171 | 4.160 | 0.541 | 32.016 |
| C-KIT mutations |  |  |  |  |
| Exon 11 ^a^ | 1.000 |  |  |  |
| Exon 9 | 0.013 | 3.569 | 1.304 | 9.767 |
| Exon 13 | 0.068 | 8.811 | 0.853 | 91.000 |
| Exon 9+11 | 0.024 | 20.058 | 1.475 | 272.848 |
| Exon 11+13 | 0.014 | 24.161 | 1.919 | 304.165 |
| Wild | 0.858 | 0.828 | 0.106 | 6.485 |

Abbreviations: CXI, Cachexia Index; HR, Hazard Ratio; CI, Confidence Interval

^a^ Reference categories
